# Bayesian Estimation of Conditional Independence Graphs Improves Functional Connectivity Estimates

**DOI:** 10.1371/journal.pcbi.1004534

**Published:** 2015-11-05

**Authors:** Max Hinne, Ronald J. Janssen, Tom Heskes, Marcel A.J. van Gerven

**Affiliations:** 1 Radboud University, Institute for Computing and Information Sciences, Nijmegen, the Netherlands; 2 Radboud University, Donders Institute for Brain, Cognition and Behaviour, Nijmegen, the Netherlands; University of Pennsylvania, UNITED STATES

## Abstract

Functional connectivity concerns the correlated activity between neuronal populations in spatially segregated regions of the brain, which may be studied using functional magnetic resonance imaging (fMRI). This coupled activity is conveniently expressed using covariance, but this measure fails to distinguish between direct and indirect effects. A popular alternative that addresses this issue is partial correlation, which regresses out the signal of potentially confounding variables, resulting in a measure that reveals only direct connections. Importantly, provided the data are normally distributed, if two variables are conditionally independent given all other variables, their respective partial correlation is zero. In this paper, we propose a probabilistic generative model that allows us to estimate functional connectivity in terms of both partial correlations and a graph representing conditional independencies. Simulation results show that this methodology is able to outperform the graphical LASSO, which is the de facto standard for estimating partial correlations. Furthermore, we apply the model to estimate functional connectivity for twenty subjects using resting-state fMRI data. Results show that our model provides a richer representation of functional connectivity as compared to considering partial correlations alone. Finally, we demonstrate how our approach can be extended in several ways, for instance to achieve data fusion by informing the conditional independence graph with data from probabilistic tractography. As our Bayesian formulation of functional connectivity provides access to the posterior distribution instead of only to point estimates, we are able to quantify the uncertainty associated with our results. This reveals that while we are able to infer a clear backbone of connectivity in our empirical results, the data are not accurately described by simply looking at the mode of the distribution over connectivity. The implication of this is that deterministic alternatives may misjudge connectivity results by drawing conclusions from noisy and limited data.

## Author Summary

Significant neuroscientific effort is devoted to elucidating functional connectivity between spatially segregated brain regions. This requires that we are able to quantify the degree of dependence between the signals of different areas. Yet how this must be accomplished—using which measures, each with their own limitations and interpretations—is far from a trivial task. One frequently advocated metric for functional connectivity is *partial correlation*, which is related to conditional independence: if two regions are independent, conditioned on all other regions, then their partial correlation is zero, assuming Gaussian data. Here, we use a probabilistic generative model to describe the relationship between functional connectivity and conditional independence. We apply this Bayesian approach to reveal functional connectivity between subcortical areas, and in addition we propose different variants of the generative model for connectivity. In the first, we address how a Bayesian formulation of connectivity allows for integration with other imaging modalities, resulting in data fusion. Secondly, we show how prior constraints can be incorporated in our estimates of connectivity.

## Introduction

In the early days of neuroscience much attention was devoted to identifying the functional specialization of different brain areas [1]. More recently, this focus has shifted towards revealing how these areas are organized into networks and how these networks, rather than their individual constituents, are related to cognition [2–4] and neurological or psychological pathology [5–7]. The increasing interest in neuronal connectivity sprouted its own subdiscipline known as *connectomics* [8–10]. Within connectomics, one distinguishes between structural connectivity and functional connectivity. Structural connectivity is concerned with the anatomical white-matter fiber bundles that connect remote regions of the brain. It may be estimated in vivo by diffusion weighted MRI (dMRI), which measures the fractional anisotropy of the diffusion of water molecules [11]. Functional connectivity in turn expresses the (degree of) dependency between the neuronal activity of separate brain regions [6, 12] and is typically measured non-invasively via either functional MRI, electro- or magnetoencephalography (fMRI, EEG and MEG, respectively) [13].

Several measures to quantify (the degree of) functional coupling exist [14, 15], of which the most prevalent is covariance. When the activity signal is normalized to have zero mean and unit variance, covariance coincides with Pearson correlation. As the correlation matrix is easy to compute, it has become the de facto standard in operationalizing functional connectivity. It does however have an important drawback: it is unable to differentiate between direct and indirect effects. For example, if regions A and B are correlated, and similarly B and C show correlation, then correlation between A and C is induced [16, 17]. This poses a problem for functional connectomics, as it introduces type 1 errors. The problem may be remedied to some extent by using *partial* correlations instead. Its interpretation is similar to Pearson correlation, but it captures only direct effects as the influence from other regions is partialled out. In practical terms, the matrix of partial correlations may be obtained by taking the inverse of the covariance matrix, known as the precision matrix, and rescaling this. Assuming the data are normally distributed, both the precision matrix and the partial correlation matrix capture the conditional independence structure of the considered variables, i.e. when two regions are conditionally independent given all other regions, their precision and partial correlation are zero.

Ideally, the partial correlation matrix would correctly reflect the functional connectivity that generated the observed data. If this matrix is sparse, the corresponding conditional independence graph provides an intuitive representation of the interaction between different regions. In practice however, the obtained partial correlation matrices are not sparse, which makes the estimated connectivity more difficult to interpret. In addition, if the number of samples is small and the number of regions large, there is no unique inverse of the covariance matrix and consequently no unique matrix of partial correlations. Even when these conditions are met, the maximum likelihood solution is often ill-behaved, in which case the solution must be regularized [18]. A popular approximation of the precision matrix is acquired via the graphical LASSO (Least Absolute Shrinkage and Selection Operator), which regularizes the elements of the precision matrix using the *ℓ*_1_-norm [14, 16, 19]. This approach shrinks the partial correlations towards zero so as to create sparse solutions, which are easier to interpret. Although the graphical LASSO was found to be one of the must accurate methods in identifying connectivity in a comparative study [14], it introduces a bias that underestimates functional connectivity, thus creating type 2 errors [20]. In addition, both the original maximum likelihood solution as well as the LASSO estimate provide point estimates that do not quantify the reliability of their outcome. In earlier work, we have proposed a Bayesian alternative to the graphical LASSO that uses the *G*-Wishart distribution to restrict the partial correlation estimates to a previously defined conditional independence graph. We showed that structural connectivity provides an elegant candidate for this graph, and that this approach was able to outperform the graphical LASSO on simulated data [20]. Importantly however, we assumed that the conditional independence graph was available a priori. In the current contribution we take this line of reasoning a critical step forwards and learn both functional connectivity as well as its conditional independence structure simultaneously. Apart from estimating the degree to which two regions have correlated activity, we can now also express the probability of these regions being conditionally independent. As we will show, this results in a more effective approach to regularization than the graphical LASSO, while retaining the additional benefits of the Bayesian framework.

At the foundation of this contribution lies a probabilistic generative model that describes how a particular independence structure generates partial correlations that in turn generate observable data. Using a neurologically plausible simulation with several different conditions, as described by Smith et al. [14], we show that in many cases our Gaussian graphical model approach is favorable to both the maximum likelihood alternative and graphical LASSO regularized solutions. Subsequently, we apply the model to estimate functional connectivity between bilateral accumbens, amygdala, caudate, hippocampus, pallidum, putamen and thalamus using their blood-oxygenation level dependent (BOLD) signal time courses, measured using resting-state fMRI. Finally, we demonstrate how the advantages of a Bayesian approach can be put to practice by showing two extensions to our connectivity model. First, we show how the problem of data fusion for connectivity studies [21, 22] may be tackled by simply providing multiple likelihood terms; one for each imaging modality. This is demonstrated empirically by combining the fMRI time series with dMRI probabilistic tractography results. Second, we describe how further background knowledge on putative connections may be used to both constrain and inform functional connectivity.

## Methods

### Functional connectivity as a Gaussian graphical model

From a methodological perspective, elucidating functional connectivity is often rephrased as a covariance selection problem. This boils down to finding a sparse partial correlation matrix associated with the time series (activity) of a set of variables (brain regions), a problem known as covariance selection. Here, the problem is approached using a Gaussian graphical model (GGM), where we assume that the data **X** = (**x**_1_, …, **x**_*n*_)^*T*^ consist of *n* independent draws from a *p*-dimensional multivariate Gaussian distribution $N(0,K^{-1})$, with zero mean and precision (inverse covariance) matrix **K**. Here, $K\in P_{p}$, with $P_{p}$ the space of positive definite *p* × *p* matrices. The likelihood of **K** is given by

$$P\left(X|K\right)=\prod_{i=1}^{n}N\left(x_{i}|0,K^{-1}\right)\propto {\left|K\right|}^{n/2}\exp[-\frac{1}{2}\;\langle K,\Sigma \rangle ]\;,$$ (1)

where **Σ** = **X** ^*T*^ **X** and ⟨⋅, ⋅⟩ the trace inner product operator. The assumption of Gaussianity is justified empirically, as BOLD data has been shown to follow a Gaussian distribution [23].

The precision matrix has the important property that zero elements correspond to conditional independencies, provided the data are normally distributed. In other words, Eq (1) specifies a Gaussian Markov random field with respect to a graph *G* = (*V*, *E*), with *V* = {1, …, *p*} and *E* ⊂ *V* × *V*, in which the absence of a connection indicates conditional independence, i.e. (*i*, *j*) ∉ *E* → *k*_*ij*_ = 0 [24, 25].

In order to estimate the precision matrix **K** of a zero-mean multivariate Gaussian density from data **X** one may maximize the log-likelihood which gives the maximum likelihood estimate (MLE):

$$\hat{K}=\underset{K\in P_{p}}{arg max}\left(\log|K|-\left\langle \Sigma K\right\rangle \right)$$ (2)

where the maximization is constrained to precision matrices in the family of *p* × *p* positive definite matrices $P_{p}$. If **Σ** is positive-definite, there exists a unique solution to Eq (2) in the form of **Σ** ^−1^. However, if the number of samples is small compared to the number of variables, the solution does not exist, and even if *n* > *p*, the maximum likelihood estimate is often ill-behaved and requires regularization [18]. A frequently used method of regularization is called the graphical LASSO [26], which penalizes the magnitude of the elements of **K**. The LASSO approach gives the following MLE:

$$\hat{K}=\underset{K\in P_{p}}{arg max}[\log|K|-\left\langle \Sigma K\right\rangle -\lambda {∥K∥}_{1}]\;,$$ (3)

in which the shrinkage parameter *λ* determines the amount of penalization that is applied. Several studies have applied the graphical LASSO in order to estimate functional connectivity [14, 16, 19]. Alternative regularization schemes are available [27], such as ridge regression or elastic net [28], but we will not consider these methods in detail here. Rather, we emphasize that each of these regularization approaches provides only a point estimate, instead of a posterior distribution over **K**. This makes it impossible to quantify the uncertainty associated with the estimate, which can lead to incorrect conclusions about functional connectivity in light of finite data. Moreover, it has been shown that the graphical LASSO is not guaranteed to find the true graph even in the limit of infinite data [29]. In addition, solutions obtained through regularization tend to underestimate functional connectivity [20].

Recently, extensions of the (graphical) LASSO approach have been proposed that allow for statistical inference. For example, [30] introduce a significance test that can be applied to LASSO estimates while [31, 32] describe a *desparsified* LASSO that attempts to de-bias the results using a projection onto the residual space. However, these approaches make assumptions on the sparsity of **K**, which may not be warranted.

Alternatively, a Bayesian approach can be applied to the covariance selection problem, which dispenses with these assumptions. It requires that we specify a prior distribution on **K**. As we hope to identify conditional independencies between the considered variables, a convenient prior distribution arises in the form of the *G*-Wishart distribution [33]:

$$P\left(K|G,\delta ,D\right)=W_{G}\left(\delta ,D\right)=\frac{{\left|K\right|}^{(\delta -2)/2}}{Z_{G}\left(\delta ,D\right)}\exp[-\frac{1}{2}\;\langle K,D\rangle ]\;1_{K\in P_{G}}\;,$$ (4)

in which $P_{G}$ is the space of positive definite *p* × *p* matrices that have zero elements wherever (*i*, *j*) ∉ *G*, *δ* is the degrees of freedom parameter, **D** is the prior scaling matrix and $1_{x}$ evaluates to 1 if and only if *x* holds and to 0 otherwise. The *G*-Wishart distribution is conjugate to the multivariate Gaussian likelihood in Eq (1), so that

$$P\left(K|G,\delta ,D,X\right)=W_{G}\left(\delta +n,D+\Sigma \right)=\frac{{\left|K\right|}^{(n+\delta -2)/2}}{Z_{G}\left(\delta +n,D+\Sigma \right)}\exp[-\frac{1}{2}\;\langle K,D+\Sigma \rangle ]\;.$$ (5)

Note that the Wishart distribution is a special case of the *G*-Wishart distribution, with which it coincides if *G* is a fully connected graph.

It should be pointed out that in the limit of *n* → ∞, any prior will be fully dominated by the data. In theory, even when the true precision matrix **K** contains very small elements, the probability of a corresponding edge will go to 1 in the limit of an infinite amount of data. The interesting question is what happens if the magnitude of these elements scales as a function of *n*, e.g., as 1/*n*. Where asymptotic analyses have been successfully applied to better understand the behavior of regularization approaches such as the graphical LASSO [34, 35], such analyses of Bayesian procedures are complex and may lead to counterintuitive results [36]. For the *G*-Wishart prior in particular, similar analyses have, to the best of our knowledge, not yet been pursued.

**Fig 1:**
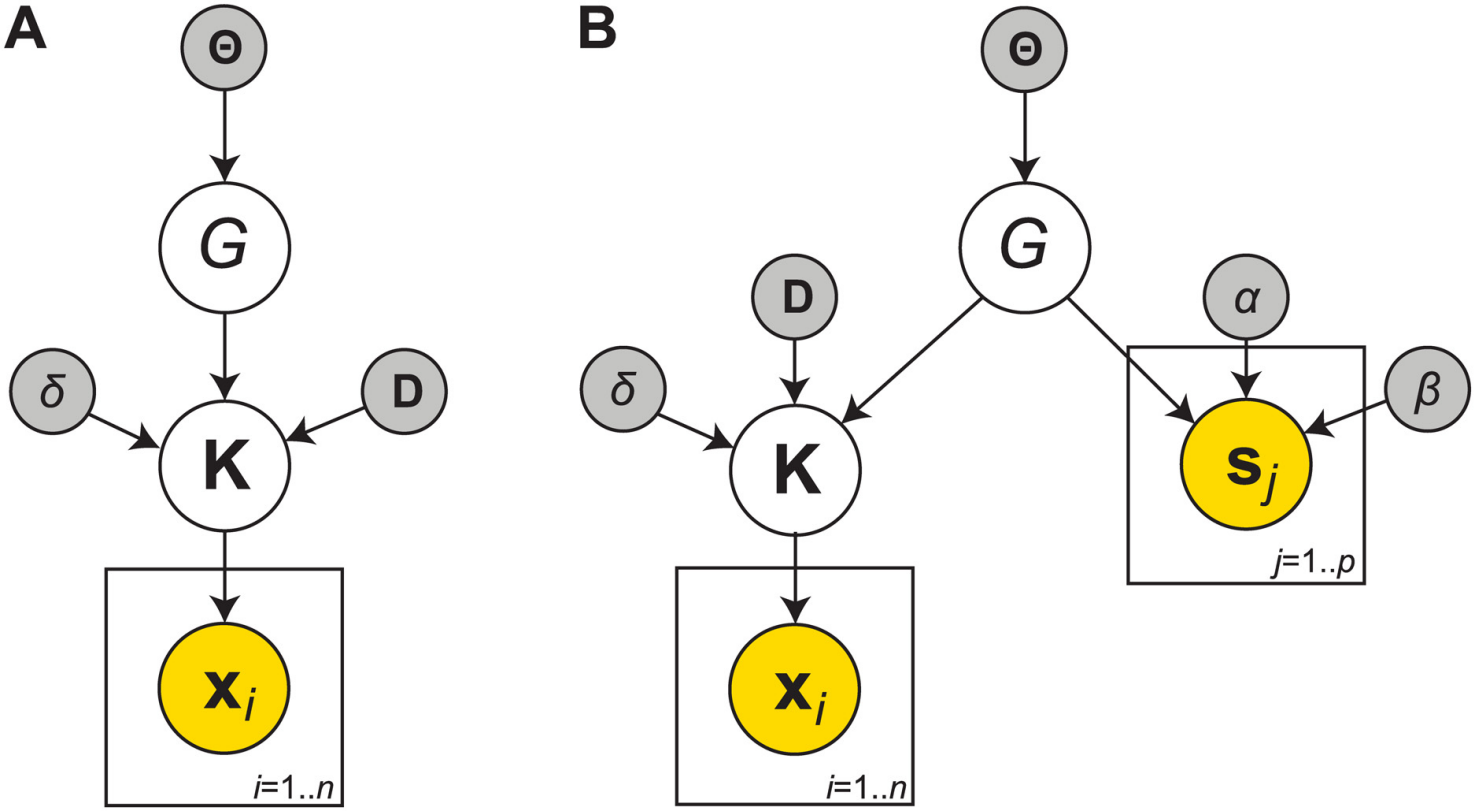
**A** The generative model for the conditional dependencies graph and precision matrix. **B** The generative model for structural connectivity and the precision matrix, based on both BOLD time series **X** and probabilistic streamline counts **N**. Latent variables, observed variables and hyperparameters are indicated in white, yellow and grey, respectively.

The preliminaries described above allow us to specify the distribution that is central to this work, i.e. the joint posterior over both the conditional independence graph and the precision matrix (an illustration of the graphical model is provided in Fig 1A):

P(G,K∣X)∝P(X∣K)P(K∣G)P(G). (6)

Note that the necessary hyperparameters are typically omitted for clarity.

In practice, functional connectivity is more intuitively understood in terms of partial correlations than as elements of the precision matrix. The partial correlation matrix **R** may be obtained from the precision matrix by applying the transformation

$$r_{ij}=\{\begin{array}{cc} 1 & \text{if}\;i=j\text{,}\;\\ -\frac{k_{ij}}{\sqrt{k_{ii}k_{jj}}} & \text{otherwise.} \end{array}$$ (7)

By transforming each element of **K** in Eq (6), the distribution *P*(*G*, **R** ∣ **X**) is constructed. When discussing our experimental results, we will focus on partial correlations rather than precision values, unless explicitly stated otherwise. Note that the relation between the dependency structure *G* and the precision matrix **K**, as discussed above, also holds between *G* and the partial correlations **R**. That is, absence of a connection in (*i*, *j*) ∈ *G* implies *r*_*ij*_ = 0.

The Bayesian generative model must be completed by specifying a prior distribution to draw *G* from. Here, we assume that a priori all edges are marginally independent and each have probability *θ*. That is, we have

$$P\left(G|\Theta \right)=\prod_{i<j}\theta_{ij}^{g_{ij}}{\left(1-\theta_{ij}\right)}^{1-g_{ij}}\;,$$ (8)

with *g*_*ij*_ ∈ {0, 1}, *g*_*ij*_ = 1 ↔ (*i*, *j*) ∈ *G* and Θ = (*θ*_*ij*_)_*i* < *j*_. Initially we use *θ*_*ij*_ = 0.5 ∀_*i*, *j*_ to indicate that we have no a priori preference for either a dependence or an independence. The impact of different values for *θ*_*ij*_ on the posterior estimates is discussed in S3 Text, where it is shown that the prior is to a large extent dominated by the likelihood.

### Functional connectivity variants

One of the benefits of the Bayesian framework is that extensions to the generative model are straightforward to implement. In this section we use the distribution given in Eq (6) to provide two illustrations of such extensions for analyzing connectivity.

#### Integrating additional modalities

In the ideal case where the complete neural system is considered (i.e. there are no hidden variables that may explain away some conditional dependencies), the conditional independence graph almost entirely coincides with the structural connectome as each functional relation must be facilitated by an anatomical connection [37]. In other words, *G* now represents both the conditional independence graph as well as structural connectivity. In this case, functional connectivity may be estimated more accurately by incorporating additional imaging modalities that inform the conditional independence structure. To do so, we must employ an additional likelihood term describing how the data from the extra imaging modality are generated by *G*. The posterior distribution of connectivity is then given by

$$P\left(G,K|Y\right)\propto P\left(K|G\right)P\left(G\right)\prod_{Y\in Y}P\left(Y|K,G\right)\;,$$ (9)

with Y the collection of data sets to be combined. The result of this mathematically straightforward exercise provides an elegant way to obtain data fusion. While several techniques have been proposed to achieve this (see e.g. [22, 38] for reviews on this topic), these typically rely on ad-hoc strategies instead of a generative model. Although the choice for specific probability distributions may be subject to change, the generative modeling approach serves as a generic way to link structural and functional connectivity and the different modalities that provide data regarding them.

Here, we use an existing model of structural connectivity based on probabilistic tractography [39, 40], defined as follows. The matrix **S** is assumed to contain probabilistic streamline counts [41] that run from region *j* to all other {1, …, *p*} \ *j* regions. It is generated from existing anatomical connections, i.e. structural connectivity, through

$$P\left(S|G,\alpha ,\beta \right)=\prod_{j}\text{DirMult}\left(\alpha g_{j}+\beta \left(1-g_{j}\right)\right)\;,$$ (10)

wherein *α* and *β* are hyperparameters that govern the distributions of streamlines over existing and absent connections, respectively. Integration with the Gaussian graphical model is achieved by incorporating Eqs (10) into (9):

P(G,K∣X,S)∝P(X∣K)P(K∣G)P(S∣G)P(G). (11)

A visual representation of the generative model is shown in Fig 1B. Throughout this paper we refer to our method as the Bayesian Gaussian graphical model (BGGM) approach.

#### Informative prior

The assumption that the prior probability of connections is the same for all region pairs (see Eq (8)) is rather crude, and may be replaced depending on available background information. To illustrate this, we describe an additional approach to connectivity based on the assumption that homotopic regions in different hemispheres are directly connected, but that other interhemispheric connections do not exist. Within either hemisphere, we remain agnostic about connectivity. This intuition is easily formalized by

$$\theta_{ij}=\{\begin{array}{cc} 0.5 & \text{for}\;i\;\text{and}\;j\;\text{in}\;\text{the}\;\text{same}\;\text{hemisphere,}\\ 1 & \text{for}\;i\;\text{and}\;j\;\text{homotopic}\;\text{regions}\;\text{and}\;\\ 0 & \text{otherwise.} \end{array}$$ (12)

Clearly, this prior is more restrictive than a homogeneous prior, as most of the elements corresponding to cross-hemisphere connections are now excluded. In addition, the restrictive zero probability of some of the interhemispheric connections is an extreme choice. However, we use it here to provide an example of how information regarding the absence of connections (e.g. in the case of a white-matter lesion) affects the estimates of the present connections

### Simulation

To analyze the performance of the Gaussian graphical model approach to functional connectivity, we compare our results to those presented in [14]. Here, realistic BOLD time series are generated according to the dynamic causal modeling (DCM) fMRI forward model [42], that makes use of the nonlinear balloon model [43], based on a known constructed network as its starting point. In total, 28 simulations with different parameters such as number of nodes, number of generated samples, sampling frequency and noise levels were constructed. For each simulation, 50 different time series are generated, simulating different ‘subjects’ (throughout we will refer to these pseudo-subjects as ‘runs’, to avoid confusion with the empirical data later on). The networks in the simulations were composed of 5, 10, 15 or 50 nodes and for each node between 50 and 10 000 samples were generated. For 15 of the 28 simulations, additional characteristics were introduced, such as shared input between a number of nodes, or mixing in timeseries between nodes (mimicking the effect of bad ROI definition) [14]. For the full description of the approach as well as the additional simulation parameters, we refer to the original description in [14] as well as the corresponding web page where the simulation may be downloaded (http://www.fmrib.ox.ac.uk/analysis/netsim/). In the simulation study, it was shown that using partial correlation (both maximum likelihood as well as LASSO regularized point estimates) resulted in the best (undirected) reconstructions of the ground truth. As these methods performed best, and are closely related to our approach, we use these to compare our results with.

The evaluation procedure is as follows: For each run of each of the 28 different simulations, the time series **X** of that run are used to compute *P*(*G*, **R** ∣ **X**). In addition, for each run the maximum likelihood estimate (MLE) is computed, as well as the graphical LASSO regularized point estimate using the same regularization as in [14] (i.e. *λ* ∈ {5, 100}). The quality of the reconstruction of the ground truth is quantified in three ways. Let R* be the ground truth functional connectivity that we are trying to recover and let **T** be a matrix that has 1 in its elements whenever the corresponding edge is present in the ground truth network, and 0 otherwise (ignoring directionality). Then **Γ** = ∣R* − **R**∣ gives the reconstruction error (where **R** is either a sample from *P*(*G*, **R**), or a point estimate). The total reconstruction error is $\eta \left(\Gamma \right)=\frac{2}{p(p-1)}\;\sum_{i<j}\;\gamma_{ij}$, the true positive error is $\eta_{\text{tp}}\left(\Gamma \right)=\frac{1}{N_{\text{tp}}}\;\sum_{i<j}\;\gamma_{ij}\delta_{t_{ij}\neq 0}$, where *N*_tp_ is the number of nonzero elements in the ground truth R*, i.e. the number of true present connections, and finally the true negative error is given by $\eta_{\text{tn}}\left(\Gamma \right)=\frac{1}{N_{\text{tn}}}\;\sum_{i<j}\;\gamma_{ij}\delta_{t_{ij}=0}$, where *N*_tn_ is the number of zero elements in the ground truth R*, i.e. the number of true absent connections. The indicator function *δ*_*x*_ evaluates to 1 if and only if its argument *x* holds true, and to 0 otherwise.

**Fig 2:**
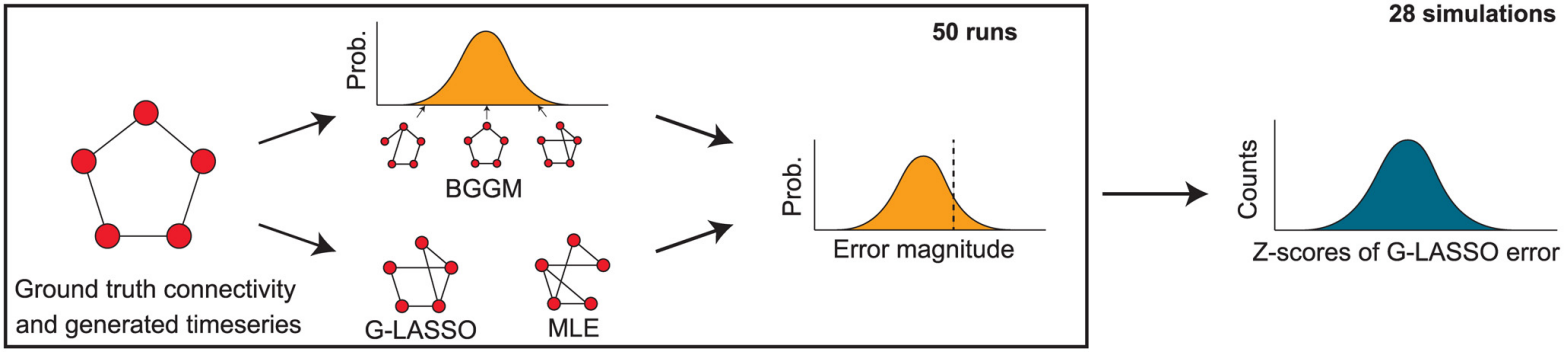
The evaluation procedure of the simulated fMRI data. First, both the posterior distribution *P*(*G*, **R** ∣ **X**) and the point estimates (for the graphical LASSO or maximum likelihood estimate) are determined. Subsequently the error compared to the ground truth is computed for all samples in the approximated distribution as well as for the point estimates (see text for this procedure). These results are summarized by computing the *z*-score for the point estimate error relative to the distribution of errors obtained from the Bayesian approach. Finally, the *z*-scores are aggregated across the runs, resulting in a histogram of error *z*-scores for each simulation.

In [14], a null distribution is computed for each of the different methods, by randomly permuting the node labels in the different runs (to remove any influence between the different nodes), which is subsequently used to derive a *z*-score for an error measure similar to *η*. However, in the case of Bayesian functional connectivity, a distribution characterizing the uncertainty of the results is already available in the form of *P*(*G*, **R**). By applying *η* to each of the samples of this distribution, we obtain *P*(*η*). The standardized scores of a point estimate **R** relative to the BGGM distribution may be computed as *z*(**R**) = (*η*(R*, **R**) − *μ*)/*σ*, in which *μ* and *σ* are the mean and standard deviation of the distribution, respectively. The procedure is illustrated in Fig 2.

### Uncertainty in connectivity distributions

The Bayesian formulation of the model allows us to describe and compare the shapes of the different posterior distributions. We compute the entropy of the posterior distributions as

$$H=-\sum_{G}\;[P\left(G,K|X\right)\log_{2}\;P\left(G,K|X\right)]\;,$$ (13)

to indicate the diversity of models that have been encountered in the Markov chains. In addition, the posterior probability of the maximum a posteriori sample is derived, i.e.

$$P\left(\hat{G},K|X\right)=\max_{G}P\left(G,K|X\right)\;,$$ (14)

to quantify how much of the posterior distribution is dominated by its mode.

### Approximate inference

The Markov chain Monte Carlo (MCMC) scheme as described in S1 Text was used to approximate the posterior distributions of interest for each subject using either the simulated BOLD signal time series, the BOLD time series data for the fourteen subcortical regions (see Eq (6)), the combination of time series data and tractography output for the subcortical regions (see Eq (11)) or finally the BOLD time series data in combination with the informed prior. Throughout, a vague prior on the precision is used: $P\left(K|G\right)=W_{G}\left(3,I_{p}\right)$, cf. [44]. The parameters of the probabilistic streamline model are set to (*α*, *β*) = (1, 0.5), which expresses that high streamline counts are most likely associated with a structural connection, while still allowing for tractography noise [40]. Once convergence was established, the approximated distributions were uniformly thinned to *T* = 1 000 samples, to make subsequent analyses more manageable and to have an equal number of samples for all different settings. Details of convergence monitoring and computation speed are provided in S2 Text.

### Materials

#### Ethics statement

Twenty healthy volunteers were scanned after giving informed written consent in accordance with the guidelines of the local reviewing committee CMO Arnhem-Nijmegen. This study was approved by CMO Arnhem-Nijmegen (CMO 2001/095 and amendment “Imaging Human Cognition”).

#### Data acquisition

The acquired data consist of a T1 anatomical scan, resting-state functional MRI data and diffusion-weighted images (DWI), collected for each subject. We refer the reader to [45] for details of the acquisition protocol. All preprocessing steps were performed using FSL 5.0 [46] with default settings unless otherwise specified.

Preprocessing of the resting-state functional MRI data consisted of the following steps. T1 images were linearly registered to MNI-152 space. Multi-echo volumes at each TR were combined [47]. Motion correction was performed using MCFLIRT and estimated motion parameters were regressed out together with their temporal derivatives and mean time courses for both WM and CSF. Finally, data were high-pass filtered at 0.001 Hz. Note that we did not apply global signal regression, as this step is known to introduce artifactual negative correlations [48, 49].

Preprocessing of the DWI data was conducted using FSL FDT [50] and consisted of motion correction, correction for eddy currents and estimation of the diffusion parameters. To obtain a measure of white-matter connectivity, we used FDT Probtrackx 2.0 [50, 51] using seed voxel to target voxel tracking. Structural scans were segmented using FAST [52] and FIRST [53] to generate seed and target voxels. Seed voxels were those voxels in the cortical gray matter mask with a non-zero white-matter partial volume estimate and the outermost voxels of the subcortical masks. The remainder of the cortical and subcortical voxels served as target voxels. In addition, streamlines were terminated once they hit the target mask. This prevents polysynaptic connections being erroneously interpreted as direct connections.

Finally, subcortical structures were segmented using FSL FIRST [53], resulting in data for a total of fourteen regions, consisting of bilateral accumbens, amygdala, caudate, hippocampus, pallidum, putamen and thalamus. For the functional data, for each of these regions the signal was averaged over all voxels in that region and subsequently standardized to have zero mean and unit variance. For the streamline data, all streamline counts were aggregated over pairs of voxels in pairs of regions, resulting in a 14 × 14 matrix of streamline counts.

## Results

Below we discuss both the simulation results as well as the connectivity estimates obtained on empirical data. For readability, we refer to the probability of conditional dependence as ‘connection probability’ and to a pair of regions that are conditionally independent or not-independent, conditioned on all other variables, simply as an ‘independent’ (or ‘disconnected’) or ‘dependent’ (or ‘connected’) region pair, respectively.

### Simulation results

**Fig 3:**
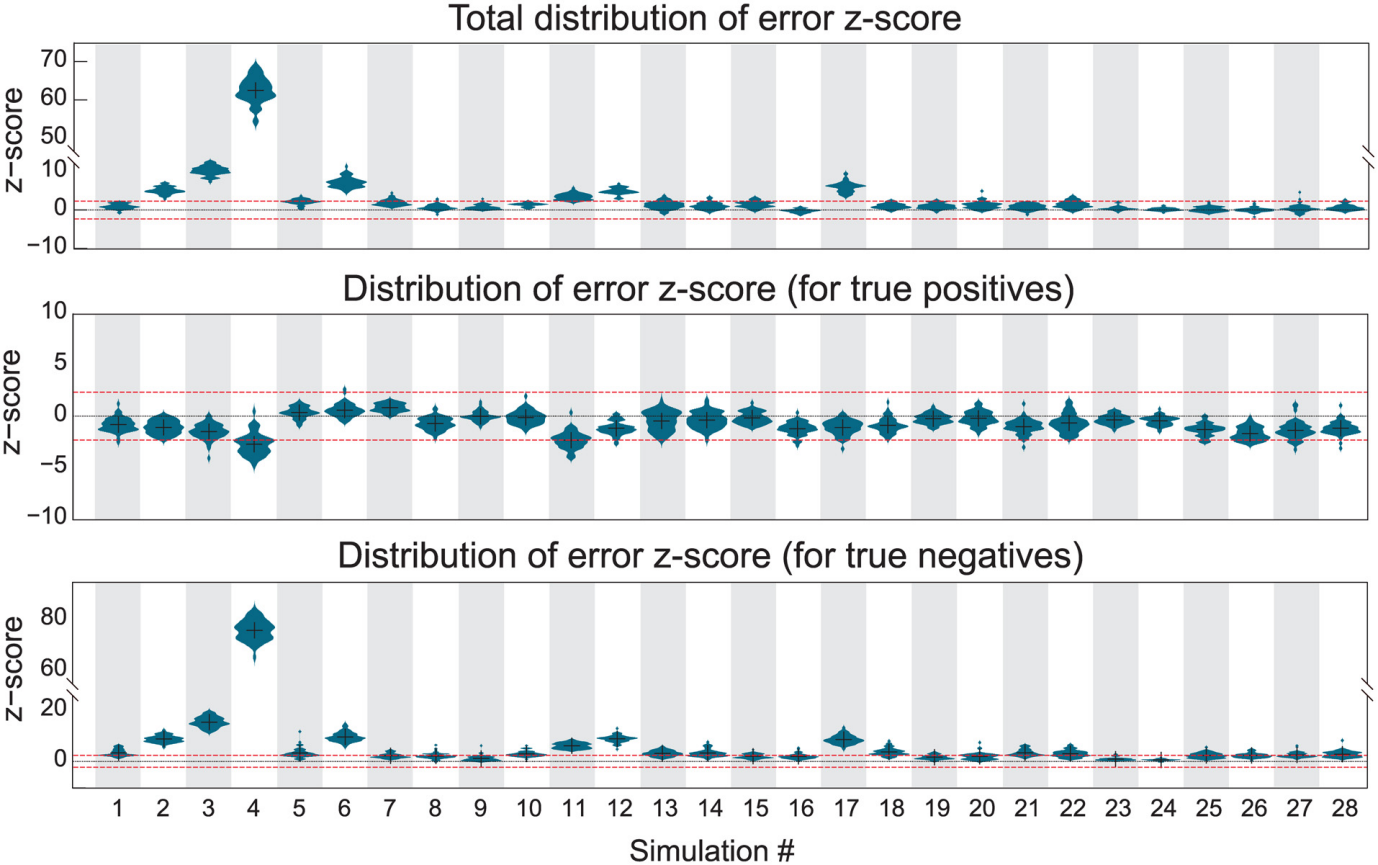
The histograms for each of the 28 different simulations. Positive error *z*-scores indicate that the point estimate was less effective in recovering the ground truth than the Gaussian graphical model, while the reverse is true for negative error *z*-scores. The red dashed lines indicate the interval outside of which the difference in performance is significant (*p* < 0.01, *z*-test). Note the different ordinate axes.

Fig 3 shows the (smoothed) histograms of *z*-scores aggregated over the 50 runs per simulation, for the graphical LASSO approach with *λ* = 100 (the results for *λ* = 5 and the MLE are almost identical; the MLE results are shown in S1 Fig). In this figure, distributions of errors with high *z*-scores have substantially larger errors than the errors from the BGGM approach, while distributions with low *z*-scores have smaller errors. The significance threshold at *p* < 0.01 is indicated by the red dotted lines. The first row of Fig 3 shows the total scores (both true positives and true negatives) for each simulation, while the second and the third row split this score into the contributions for true positive connections and true negative connections, respectively. These results indicate that in terms of true positives, the LASSO approach typically has an equal to slightly better performance than our Bayesian alternative. However, the BGGM approach identifies true negatives at least as well as *G*-LASSO, and in several cases significantly outperforms it. On the whole, the proposed method is up to par with the graphical LASSO (for *λ* ∈ {5, 100}) and the MLE, while at times outperforming them greatly.

**Fig 4:**
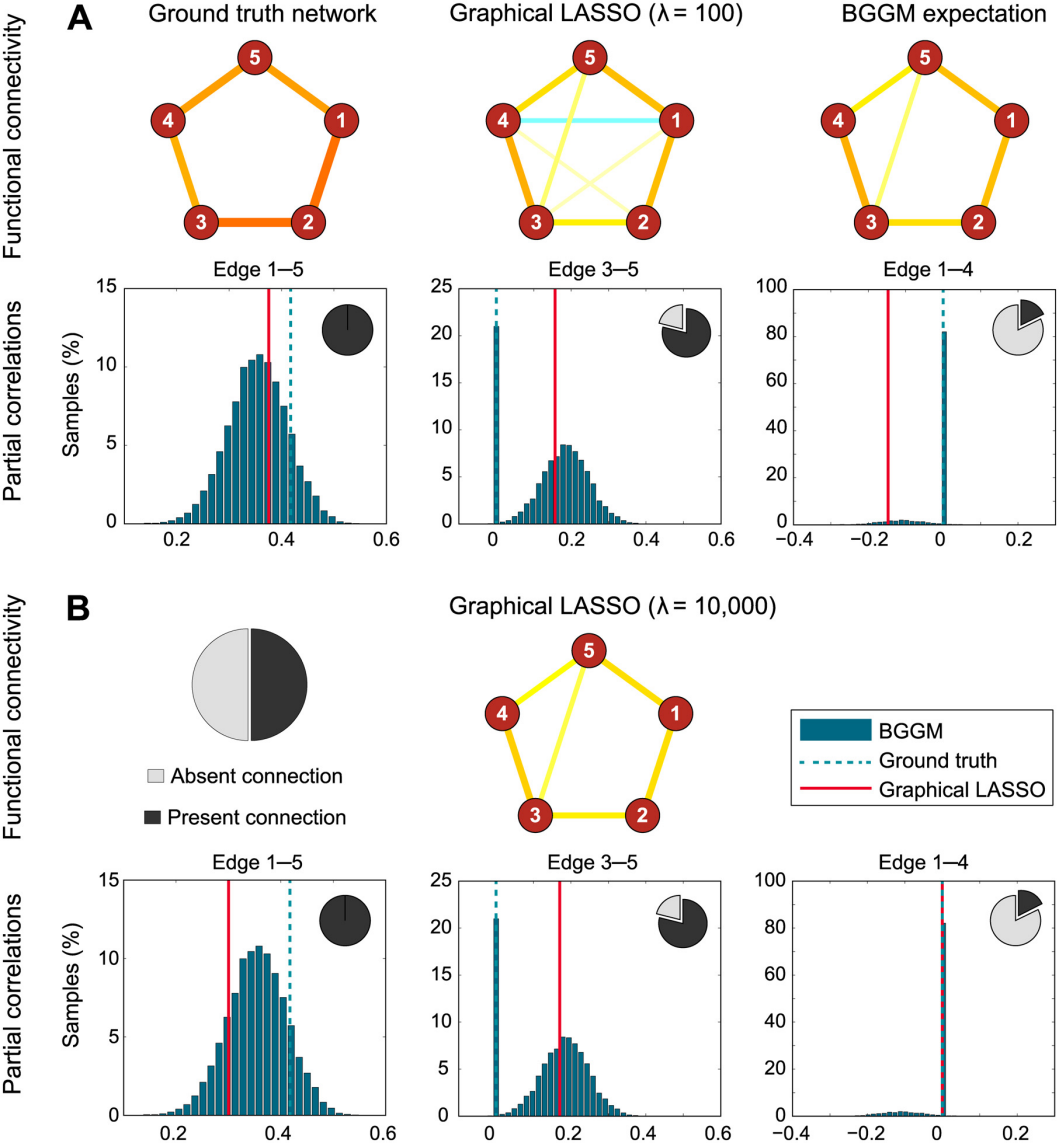
**A.** Simulation details. First row: the ground truth connectivity of one run of simulation 1, as well as the constructions by the graphical LASSO (*λ* = 100) and the expectation of the Gaussian graphical model approach. Second row: estimated partial correlation for a true positive connection, a true negative connection with strong empirical correlation, and a true negative connection with weak empirical correlation. **B.** The same, but with stronger regularization for the graphical LASSO (*λ* = 10, 000). This time, the *G*-LASSO estimate is similar to the BGGM expectation for connection 1–4, but over-regularizes the true positive connection 1–5.

To obtain insight in the behavior that creates these results, we take a closer look at some of the simulation results. As an example, Fig 4A shows the ground truth network and the reconstruction by the graphical LASSO, as well as the expectation (i.e. posterior mean of the samples) using the BGGM approach. In addition, the figure shows for three different connections the estimated partial correlation in detail. The first, between nodes 1 and 5, is present in the ground truth network. Our approximation is (correctly) confident that this node pair is not independent, and assigns a posterior partial correlation distribution close to the ground truth. The graphical LASSO estimate is slightly closer to the ground truth than the mode of the distribution. For the second node pair, between nodes 3 and 5, a connection should be absent, but because of the limited number of data samples the signals of these nodes have become correlated. This time, the BGGM approach shows a bimodal distribution. The first mode is centered close to the graphical LASSO estimate, but the second mode is at zero, as there is non-negligible evidence for this pair of nodes being disconnected. This means that on the whole (i.e. the entire distribution), the BGGM approach correctly estimates this connection strength lower than the graphical LASSO. A similar observation can be made for the third node pair, between nodes 1 and 4, of which the BGGM estimate is fairly certain about their independence. Because of this, most of the partial correlation mass is at zero, rather than at the value indicated by the graphical LASSO estimate.

These results beg the question: what if we regularize the graphical LASSO even more? Although Smith et al. report no further improvement after *λ* = 100 [14], it is possible that more regularization brings the graphical LASSO estimate closer to the BGGM results. In Fig 4B, the same visualization is provided, but this time for *λ* = 10 000. This time, we see that indeed the graphical LASSO estimate is closer to the BGGM expectation than before. In particular for the connection between nodes 1 and 4, the graphical LASSO now correctly estimates the absence of this connection. However, for the connection between nodes 3 and 5, the results hardly change, which means that the BGGM estimate is closer to the ground truth still, as, conditioned on the absent connection, the estimated partial correlation is zero. Finally, for the true positive connection between nodes 1 and 5, we see that the strong regularization causes the graphical LASSO to underestimate the connection, which will only become worse when we increment *λ* even further.

These results may similarly be interpreted in terms of the (in)dependence graph. For weak regularization, the graphical lasso suggests false positives due to limited data. For more regularization, the same dependency structure is recovered as using (the mean of) the BGGM approach (see for example Fig 4B). Regularizing even stronger introduces false negatives. Note that these results follow from the results of the recovered partial correlation structures and are therefore not explicitly presented here.

In addition, we applied the extended BIC over the ‘graphical LASSO path’ (i.e. we applied the EBIC penalty to the graphical lasso estimates over a logarithmic range of *λ*, with the maximum penalty corresponding to the empty graph, as used in [54]) to a number of simulations. However, this analysis did not result in a *λ* will results significantly different than those already presented here, and has been omitted here.

The pattern of simulations in which the BGGM outperforms the graphical LASSO is not random. In [14], each of the simulations is based on a network consisting of 5 nodes, except for simulations 2, 3, 4, 6, 11, 12 and 17, which consist of networks of 10, 15, 50, 10, 10 and 10 nodes, respectively. Precisely these simulations benefit the most from the BGGM approach, as can be seen in Fig 3. As for these simulations the ratio *N*/*p* is smallest, it is here that the most improvement can be obtained from regularization, e.g. by the graphical LASSO [14]. As we have shown above, the BGGM provides further improvement still, because this approach conditions on conditional independencies.

**Fig 5:**
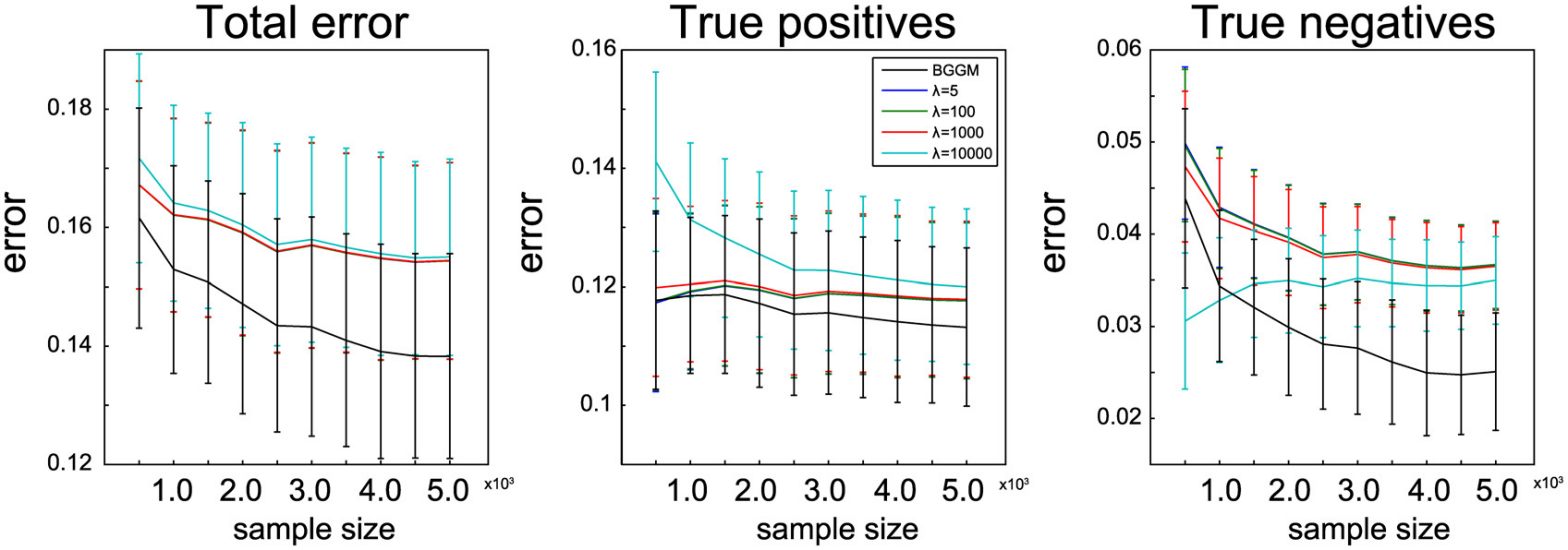
Effect of different sample sizes in recovery of ground truth connectivity, for the BGGM approach as well as for the graphical LASSO with *λ* ∈ {5, 100, 1 000, 10 000}. Error bars indicate one standard deviation over the 50 runs. For the BGGM approach, the error bars indicate one standard deviation over the expectations of the runs.

We further analyzed the effect of sample size on recovery of the ground truth by taking the simulation with the most available samples (simulation 7 in [14]) and attempting to recover the ground truth using increasingly smaller subsets of the samples. We compared the BGGM results with the graphical LASSO with *λ* ∈ {5, 100, 1 000, 10 000}. The outcome of this experiment is shown in Fig 5, once again split into the total error, error in recovery of true positives and error in recovery of true negatives. The results indicate that for small sample size, the BGGM approach already outperforms the graphical LASSO in total error, although the differences become more pronounced as more samples are considered. Extremely strong regularization (i.e. *λ* = 10 000) does result in better estimation of absent connections (by simply forcing almost all connections to zero), but this comes at the cost of excluding connections that should be present. For weak regularization (i.e. *λ* = 5), small sample size appears to be somewhat beneficial in recovery of true positive connections, as here the performance of the graphical LASSO is similar to our approach. However, this effect diminishes as more samples are acquired (inducing more spurious connections). In terms of true negatives, weak regularization is clearly outperformed by the BGGM approach.

**Fig 6:**
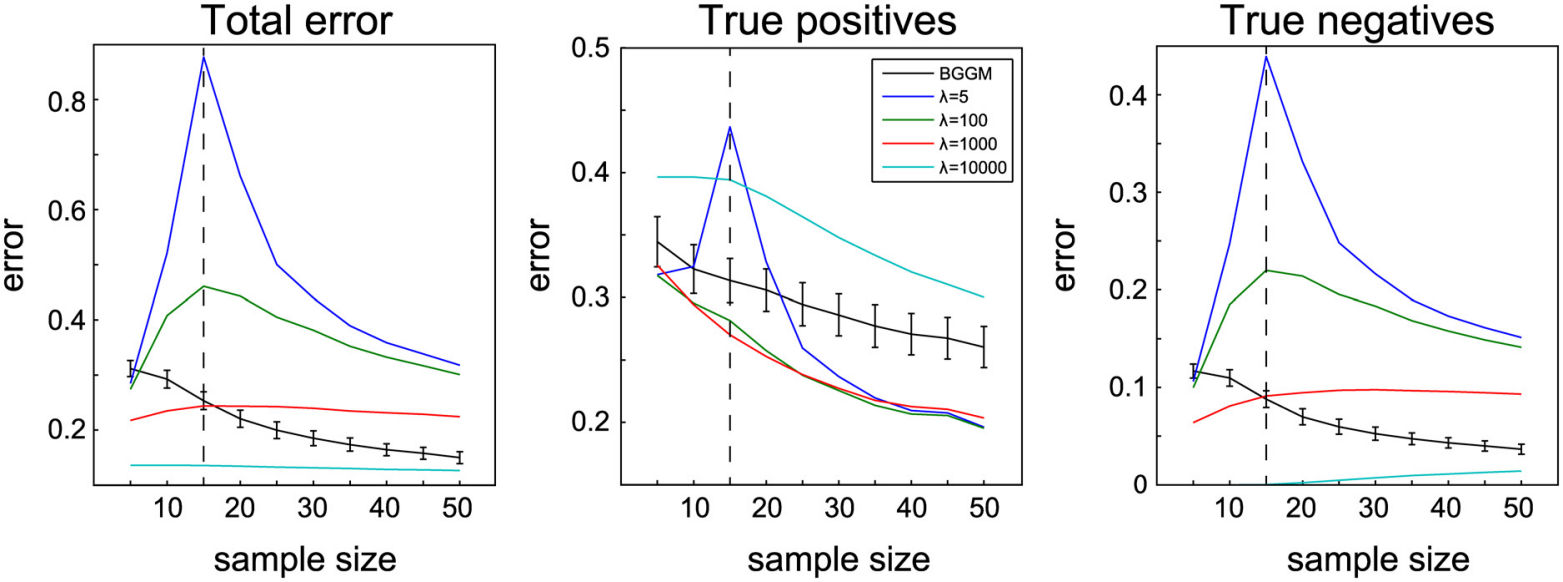
Effect of small sample sizes, including *n* < *p*, in recovery of ground truth connectivity, for the BGGM approach as well as for the graphical LASSO with *λ* ∈ {5, 100, 1 000, 10 000}. Error bars indicate one standard deviation over the 50 runs. For the BGGM approach, the error bars indicate one standard deviation over the expectations of the runs.

In addition, we analyzed the effect of small sample sizes on the estimates. We used simulation 3 (with *p* = 15) and repeated the procedure as before, but this time the number of samples was varied *n* ∈ {5, 10, …, 45, 50}, so that situations of *n* < *p* were included. The results of this experiment are shown in Fig 6. They show that, unsurprisingly, weak regularization (i.e. *λ* = 5) is insufficient to recover the ground truth when few samples are available. Strong shrinkage (i.e. *λ* = 10 000) results in a low recovery error, but this comes at the expense of significantly underestimating true positive connections. In general, the BGGM approach performs approximately equal to the graphical LASSO for small to moderate regularization, given this limited sample size scenario.

### Empirical results

Below we discuss the connectivity estimates we obtained on the empirical data, for the original BGGM model, the data fusion variant and the effect of incorporating background information.

#### Functional connectivity distributions

For all twenty subjects, functional connectivity was estimated as the posterior distribution over conditional independence graphs and partial correlation structures. We find that there is minor inter-subject variability in the number of identified non-independencies, as indicated by a small standard deviation of the mean expected density across subjects, of 0.62 (*SD* = 0.04).

**Fig 7:**
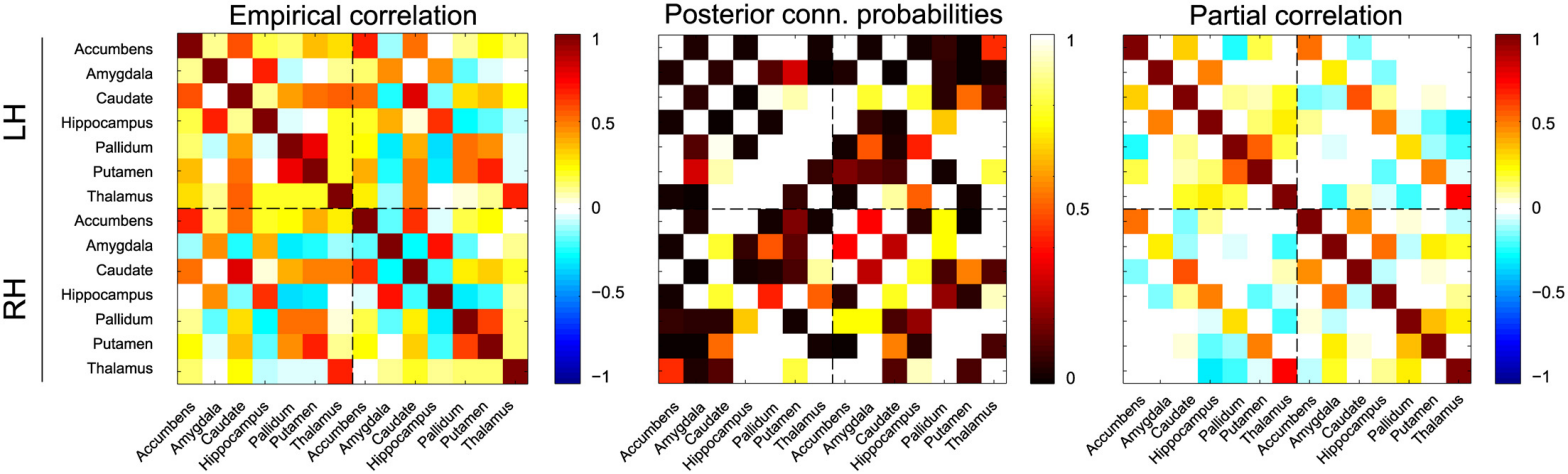
Subcortical connectivity for one subject. From left to right: the empirical correlation matrix, the mean posterior connection probability matrix and the mean posterior partial correlation matrix. The connections for the left hemisphere (LH) and the right hemisphere (RH) are separated by the dashed lines.

For the subject with the sparsest dependency structure, its mean posterior conditional independence graph as well as its mean posterior partial correlations are shown as adjacency matrices in Fig 7. The conditional independence graph for this subject has a mean density of 0.55 (*SD* = 0.03). From Fig 7 it can be seen that a number of connections are present, while supporting a partial correlation close to zero. Most likely, these connections support dependencies that are induced by noise in the data, rather than true connections between subcortical regions. This is further supported by looking at the (variance of the) group-averaged results: S2A Fig shows the group-average of the mean posterior connectivity estimates for all subjects. This reveals that no pairs of regions can consistently be marked as independent. However, a stable backbone of connections that are clearly dependent exists within both hemispheres, consisting bilaterally of accumbens—caudate, amygdala—hippocampus, pallidum—putamen, caudate—thalamus and hippocampus—thalamus, that each have a mean posterior connection probability of ≥ 0.94 and partial correlations in the range [0.15, 0.58]. Similarly, a number of connections appear stable between hemispheres. Interhemispheric connectivity consists predominantly of connections between functionally homologous regions, which have mean posterior connection probabilities of ≥ 0.95 and partial correlations in the range [0.35, 0.73]. Other strong interhemispheric connections with probability ≥ 0.90 consist of left amygdala—right hippocampus, left caudate—right thalamus, left hippocampus—right putamen, left accumbens—right caudate and left caudate—right accumbens, all with negative partial correlations in the range [−0.23, −0.16], mimicking the structure found within the hemispheres.

The between-subject standard deviation of the mean posterior estimates, as shown in S2B Fig, shows that although there is quite some between-subject variability in terms of conditional independencies, the partial correlation structures are very stable. This indicates that the Bayesian Gaussian graphical model approach explores many dependencies in the data, which can vary across subjects but contribute little to the overall partial correlation structure as they correspond to small partial correlations.

#### Bayesian data fusion

**Fig 8:**
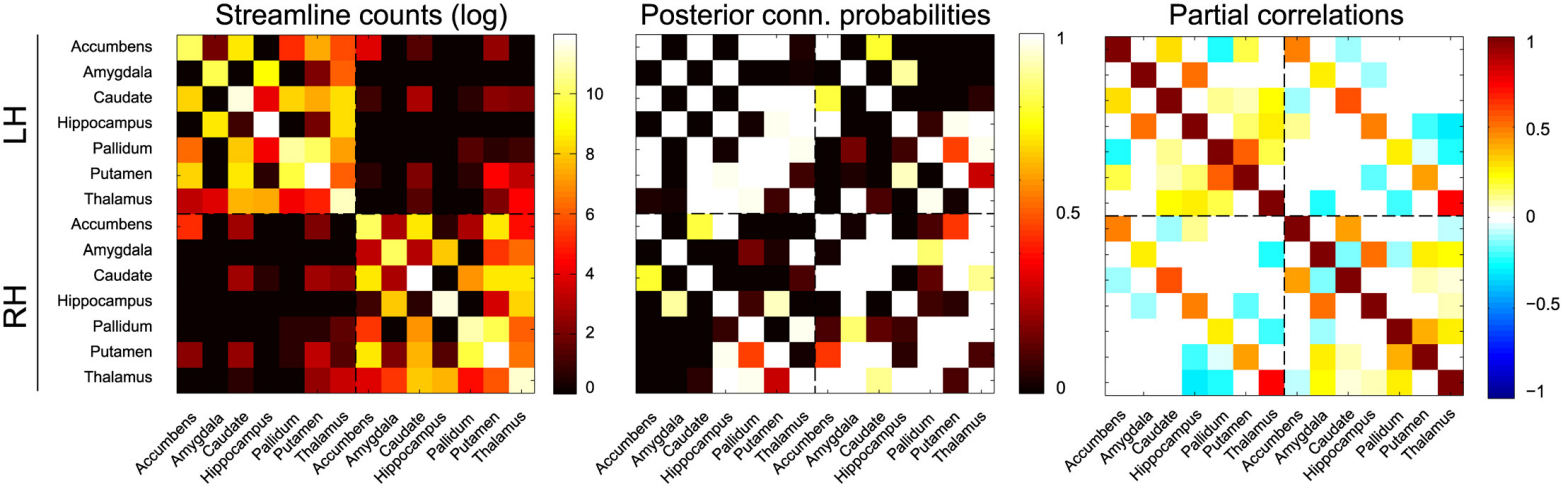
Subcortical connectivity for one subject using the data fusion model. From left to right: the empirical streamline log-counts, the mean posterior connection probability matrix and the mean posterior partial correlation matrix. Note the reduction in connectivity, in particular between the hemispheres, compared to Fig 7. The connections for the left hemisphere (LH) and the right hemisphere (RH) are separated by the dashed lines.

**Fig 9:**
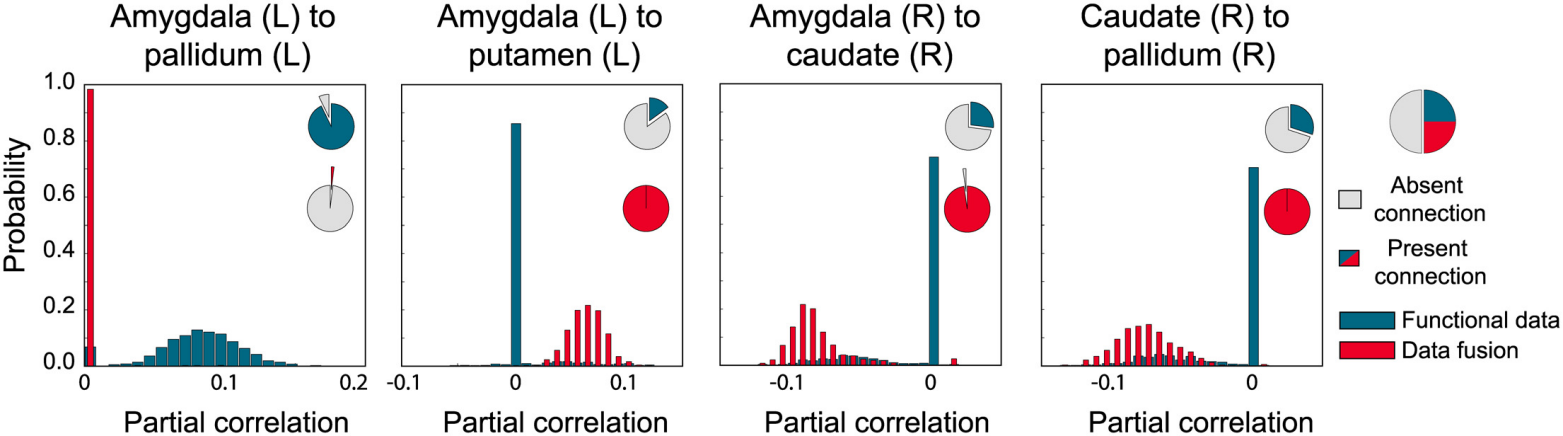
Examples of differences in partial correlation estimates between the BGGM estimates and the data fusion approach.

Similar to the previous section, functional connectivity was again estimated for all twenty subjects, but this time using the data fusion approach. This implies that the conditional independence graph is now interpreted as an estimate of structural connectivity, informed by both resting state fMRI as well as probabilistic tractography. In Fig 8, the adjacency matrices of the mean posterior estimates are shown for the same subject as used previously. Overall, the same backbone of functional connectivity is visible as when using only the fMRI data. However, there are a number of differences. In particular, adding information from probabilistic streamlines leads to substantially sparser mean network density: for this subject the density drops to 0.46 (*SD* = 0.02). In addition, particular connections change from predominantly absent to predominantly present, and vice versa. Fig 9 shows for this subject some of the connections with the largest difference in mean posterior partial correlation. This indicates that the addition of tractography data can both add and remove connections. In general however, we see that the dependencies that are removed due to the addition of tractography data, are those that supported small partial correlations.

In S3 Fig, the aggregated connectivity results are shown for all twenty subjects, as well as the standard deviations of these estimates. This reveals that the uncertainty about the retrieved connectivity decreases by adding the tractography data. Interestingly, although the expectations of the partial correlation estimates hardly change compared to the previous model (compare e.g. Figs 7 and 8), the variance of these estimates does decrease. Most likely, this is due to the fact that the bimodal behavior of partial correlations (as was observed in the simulation, where one mode is present for *g*_*ij*_ = 1 and one for *g*_*ij*_ = 0) becomes unimodal as the tractography data gives more stringent estimates of *G*.

#### Incorporating background knowledge

**Fig 10:**
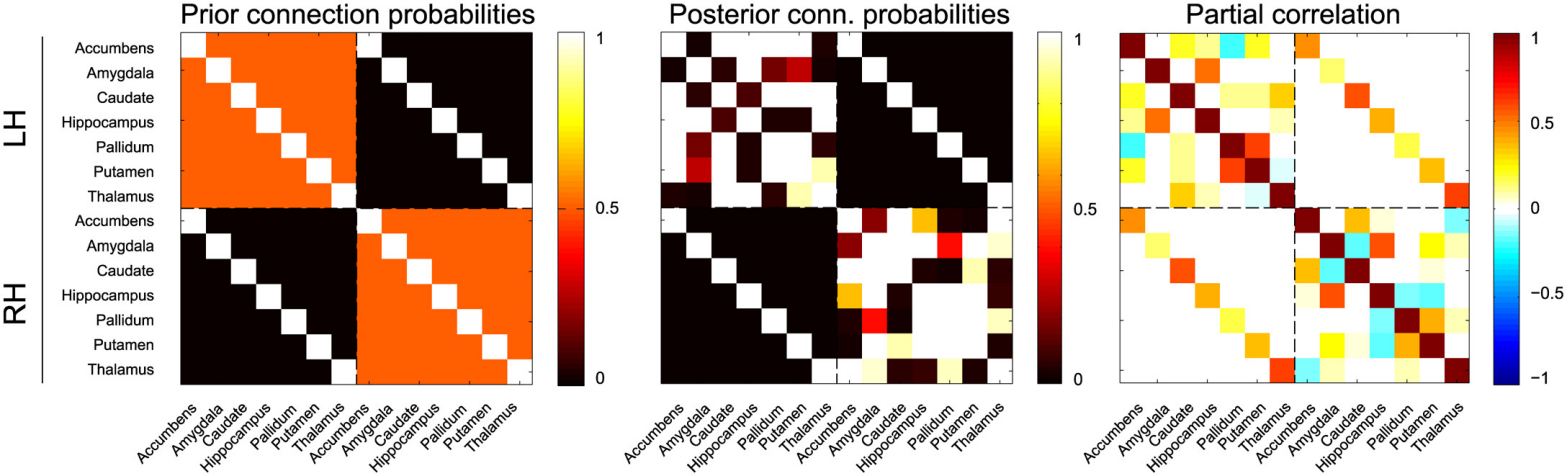
Subcortical connectivity for one subject using the informative prior. From left to right: the prior probability of a non-independence, the mean posterior connection probability matrix and the mean posterior partial correlation matrix. The connections for the left hemisphere (LH) and the right hemisphere (RH) are separated by the dashed lines.

Here we discuss the effects of assuming a priori that interhemispheric connectivity must follow the connections between the functionally homologous regions. As this prior restricts interhemispheric connections even more than the data fusion model, the network densities decrease even further. For the subject that was used as an example earlier, the connectivity matrices are shown in Fig 10. The connection density drops to 0.34 (*SD* = 0.02). Of course, this follows directly from the definition of the prior, that simply excludes a number of connections. Because of this absence of interhemispheric connections, dependencies between regions in different hemispheres must now follow a longer path via the homotopic connection. As a consequence, some of the intrahemispheric connections have an increased probability of being dependent, which we quantify by considering the density within hemispheres only. Aggregated over all subjects, we find that using the prior results in a mean density within hemispheres of 0.66 (*SD* = 0.07), slightly higher than for the initial model that has a mean density within hemispheres of 0.63 (*SD* = 0.05). The aggregated results as well as their standard deviations are shown in S4 Fig. This further shows that, similar to the data fusion model results, the variance of the elements *within* hemispheres is decreased as well, by restricting the connectivity *between* hemispheres.

#### Comparing the different distributions

**Fig 11:**
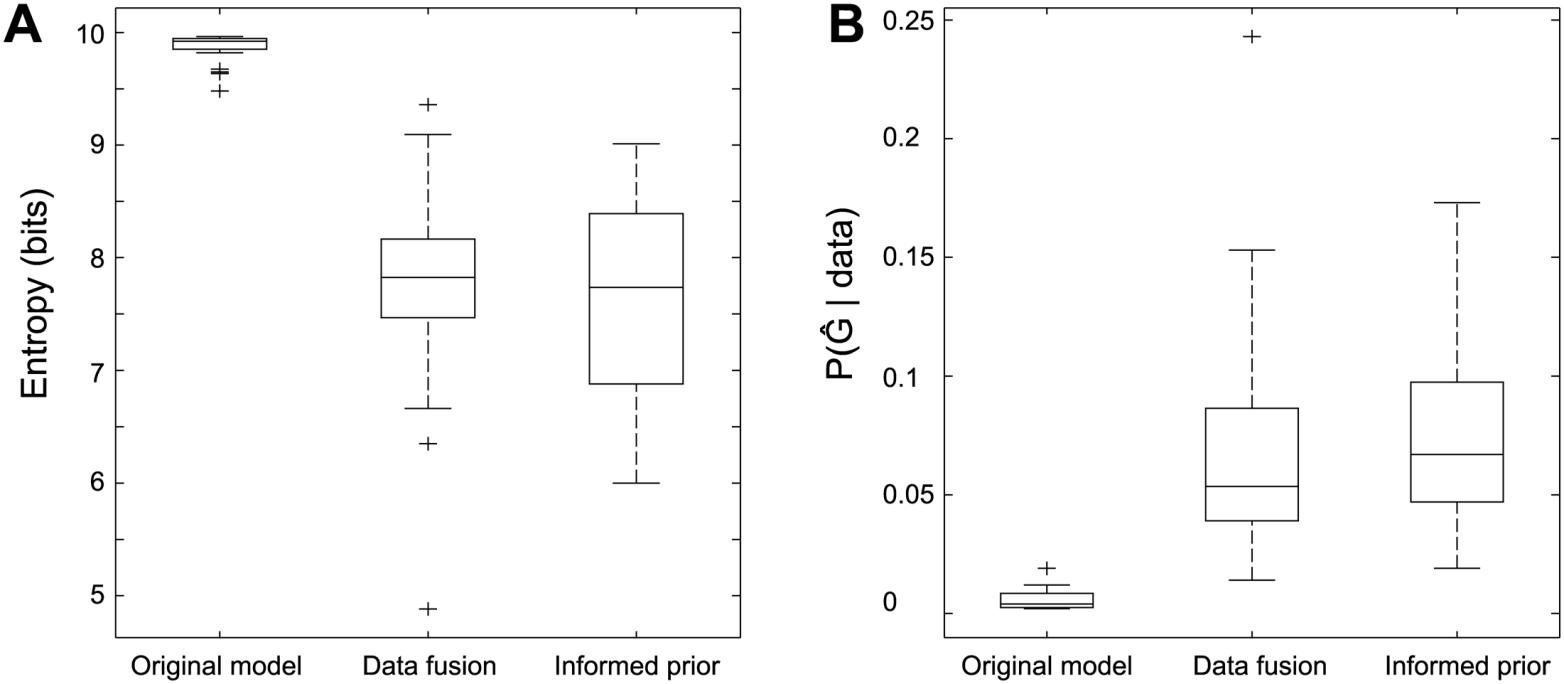
Differences in posterior distribution shapes. **A.** Entropy of the posterior distribution. **B.** Posterior probability of the mode $\hat{G}$. We refer to the prior distribution defined in Eq (8) as the vague prior.

Both the data fusion model as well as the usage of the informed prior pose restrictions on the posterior distribution of connectivity. This effect is illustrated by computing the entropy of the different approaches, as shown in Fig 11. Whereas for the original model the posterior distribution appears very broad, both alternative specifications decrease this uncertainty. In particular for the data fusion approach, one subject has a maximum a posteriori estimate with probability as high as 0.24, compared to only 0.02 when using only fMRI data. A similar picture arises by counting the fraction of unique models in each of the distributions. Here, we see that the original model has its probability density spread across many independency structures (96% ± 5 of the visited samples are unique), while the extended models are more peaked around a few high probability samples (46% ± 13 and 45% ± 13 of the samples are unique).

**Fig 12:**
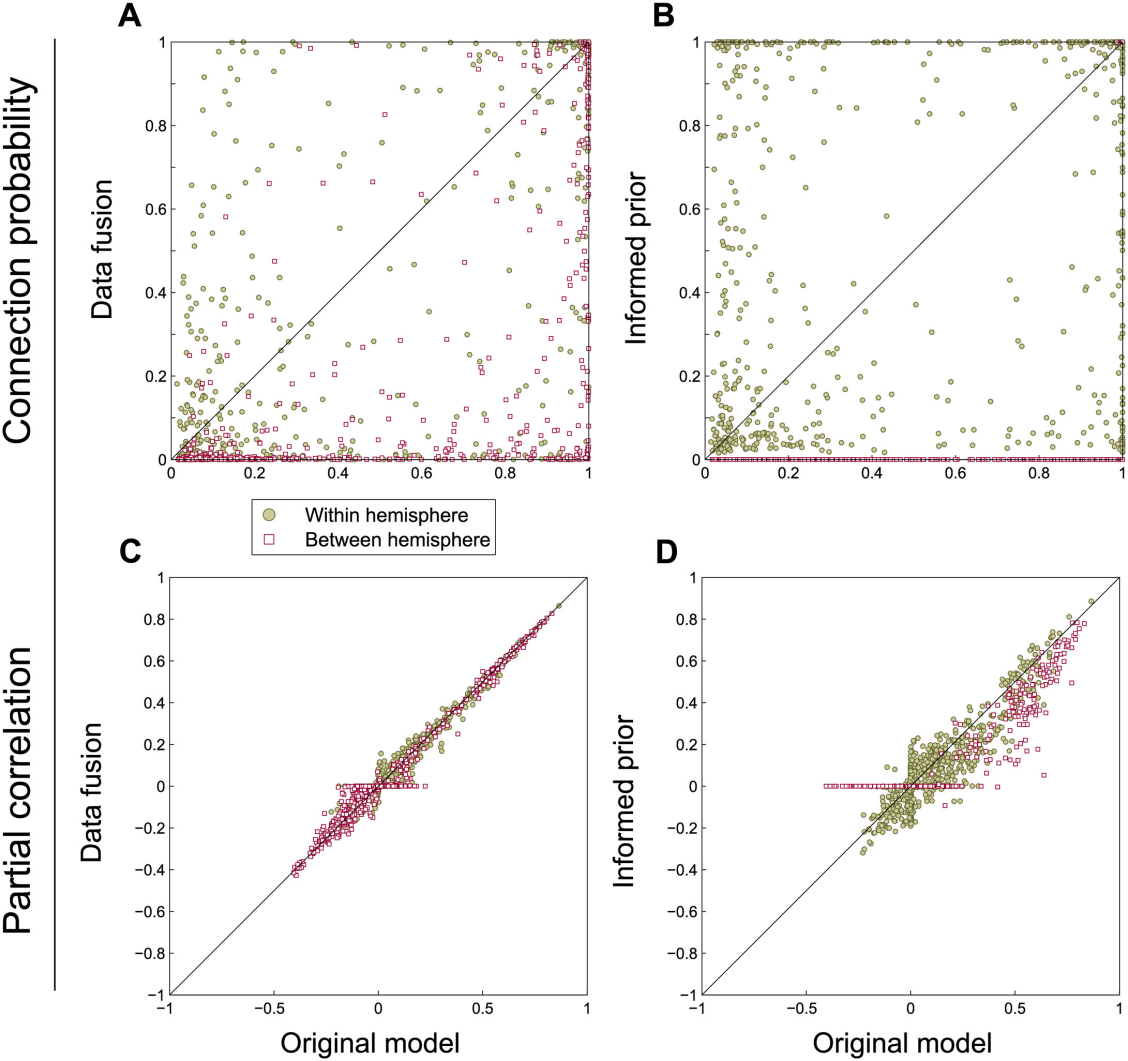
Scatter plot of the expectations of connection probabilities and partial correlations. The top row shows the connection probabilities for the two model extensions versus the original model. The bottom row shows the same, but for partial correlations.

The differences between the three approaches to connectivity are further illustrated by the scatter plot in Fig 12. Here, for all connections across all subjects the expectations of the original model compared to the two extensions are shown. Fig 12A shows that data fusion results in decreased connectivity between hemispheres. The latter connections may be less likely in the alternative model, but are not forced to zero. Partial correlations remain largely unaffected, as shown in Fig 12C, except for a few interhemispheric connections that become excluded by the tractography data and therefore are assigned zero partial correlation. The informed prior puts all interhemispheric connections to zero, as seen in Fig 12B, except for the homologous connections that have probability close to one in both models. Out of the two extensions, this approach has the most influence on the partial correlation results, as evidenced by Fig 12D. Here, not only are the interhemispheric partial correlations that do not correspond to homotopic connectivity set to zero, most other connections have lower partial correlations. This suggests that the partial correlations that are present in the original approach must be compensated by other, stronger, connections, which is no longer necessary with this prior.

**Fig 13:**
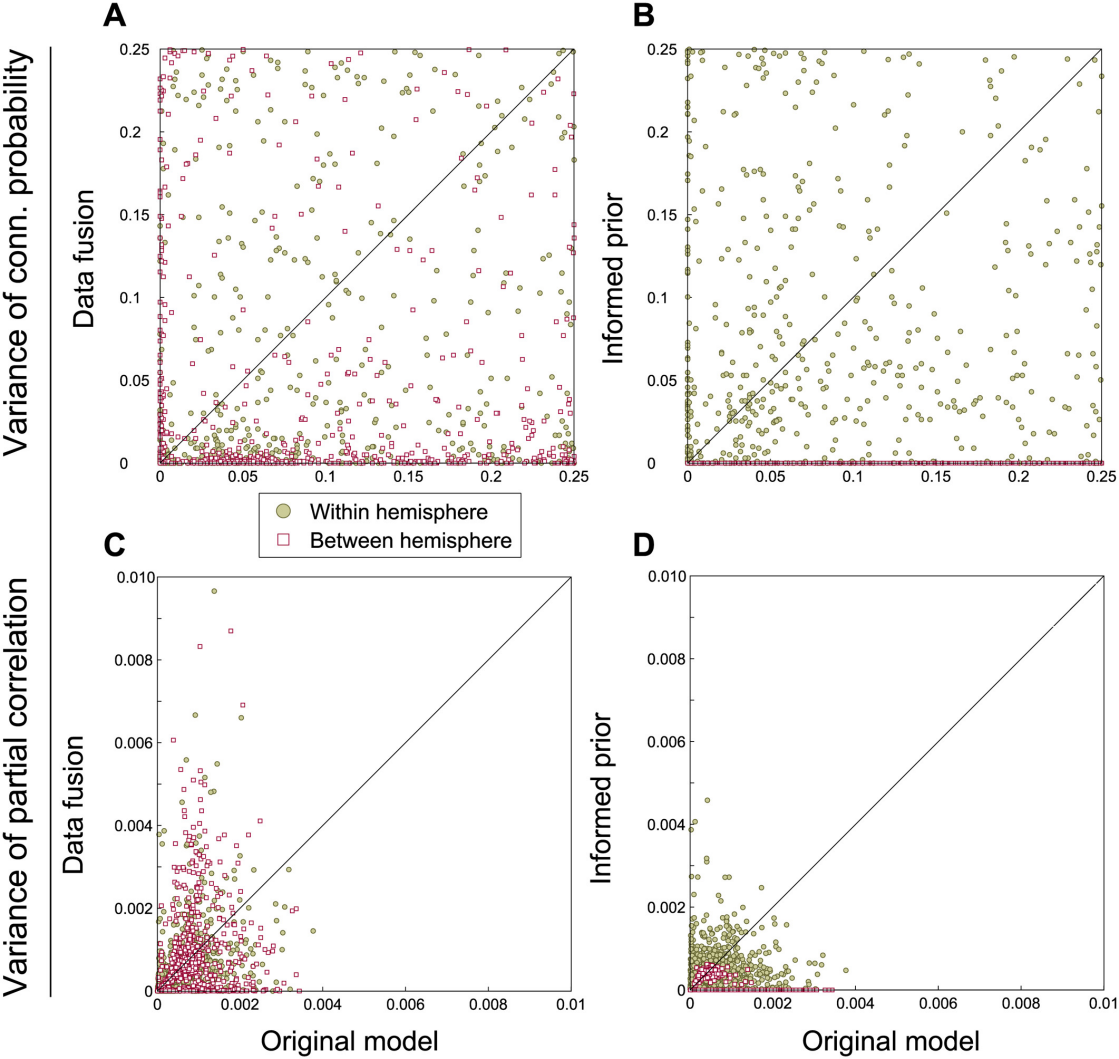
Scatter plot of the variances of connectivity and partial correlations. The top row shows the variance of connections for the two model extensions versus the original model. The bottom row is the same, but for the variance of partial correlations.

In Fig 13, the variance of the connection probabilities and partial correlations is shown. In the data fusion approach, some of the connections and partial correlations become much more precise, as shown by a lower variance (typically those connections for which no streamline data are present and which, as a result are excluded). Simultaneously, some partial correlations in fact have a larger variance (see Fig 13C), which indicates that for these connections the BOLD time series and the probabilistic streamlines contradict one another. Lastly, the informed prior obviously decreases the variance for interhemispheric connections, both in connectivity and partial correlations. For the intrahemispheric connections (about which the prior is the same as in the original model), the variance of both connectivity and partial correlations appears to remain largely unaffected. The variance of partial correlations for the connections between functional homologues decreases marginally, as shown by a mean variance of 4.0*e*−4 compared to 4.9*e*−4 for the original functional connectivity model.

## Discussion

Functional connectivity may be quantified using different metrics. The most obvious approach is to use Pearson correlation, but this metric is sensitive to polysynaptic influences. An alternative that does not suffer from this drawback is partial correlation, which was further advocated for its ability to retrieve true connections and its capacity to deal with noise [14]. Partial correlation between two variables may be interpreted as Pearson correlation conditioned on all other variables. In practice, partial correlation can be computed by applying a simple transformation to the precision matrix of a multivariate Gaussian distribution. The precision matrix and, consequently, the matrix of partial correlations, has the interesting property that conditional independence between variables, given all other variables, appears as a zero value in the corresponding matrix element [20, 55], which may conveniently be collected in a conditional independence graph. Typically, this graph is mostly ignored, while the precision or partial correlation matrix is considered the quantity of interest. In this paper, we have provided a Bayesian generative model for functional connectivity in which the conditional independence graph plays a central role, as it is assumed to generate the precision matrix and thus functional connectivity. As opposed to regularized maximum likelihood estimates for the precision matrix, our approach characterizes the full posterior distribution of both conditional (in)dependencies and partial correlations. In addition to this model, we described a number of model variants that address specific issues with, and conceptual extensions to, connectivity.

We subjected our approach to the simulations that were presented in [14], and compared its performance to the maximum likelihood estimate as well as to the graphical LASSO. The latter of these two has been shown to be the most successful in recovering connectivity in these simulations [14]. The results of the simulation are encouraging. Although we observe that for true positive connections, our approach occasionally underestimates connections, it more than compensates for this in correctly estimating true negatives (i.e. the sparsity structure of the network). When true positives and true negatives are both taken into account, corrected for their respective numbers of occurrence, we find that our approach performs at least as well as the graphical LASSO, and significantly better for simulations with small sample size compared to the number of nodes in the network. A closer look at these results shows that when estimating partial correlations, conditioning on the presence or absence of a connection provides a considerable advantage over shrinkage. In particular for connections with a moderate probability of independence our method yields a bimodal distribution of partial correlations, differentiating between the conditionally dependent and independent node pairs.

In addition to our simulation results, we used our approach to approximate the posterior distribution of functional connectivity between subcortical areas for twenty participants. This allowed us to identify a connectivity backbone that consists of strong connections and partial correlations. At the same time, we see that a number of connections are strongly dependent, but foster only weak partial correlations. This emphasizes that a richer picture of connectivity is obtained by looking at both the structure of conditional independence, as well as the strength of these connections in terms of partial correlation.

Partial-correlation based methods are susceptible to common input effects that may induce spurious connections if they are not accounted for, for example when variables (i.e. brain regions) are missing [56, 57] from the analysis. If instead the full neural system is observed, it is straightforward that direct functional connections presuppose anatomical connections between the corresponding regions. This allows us to combine the generative model for functional connectivity with a similar model for structural connectivity [39] using probabilistic tractography obtained from diffusion weighted MRI. Conceptually, this results in a data fusion model in which an underlying model of anatomy drives both the observations for functional interactions, as well as for estimates of structural fibres. Compared to alternatives that, for example, weigh a regularization parameter by the strength of structural connectivity [22, 38, 58–61], our approach is based on a generative model in which data fusion is made possible by the use of different likelihood terms. Furthermore, in our model both sources of data affect both types of connectivity; structural connectivity regularizes functional connectivity and simultaneously functional dependencies influence the probability of structural connections. On empirical data the data fusion approach leads to sparser connectivity, in particular between hemispheres. However, some connections are conditionally dependent to such a degree that the model infers a connection regardless of the lack of support by the tractography data. This is helpful in estimating structural connectivity, as it is well known that structural connectivity based on diffusion weighted imaging suffers from a large number of false negatives [62]. In addition, data fusion lowers the variance for many of the partial correlations, indicating that combining both imaging modalities leads to more robust estimates [49, 62–64]. However, for a number of connections the data for functional and structural connectivity appear to contradict each other, which actually results in increased variance. Note that our data fusion approach has similarities to linked ICA [65], which also uses a Bayesian generative model to integrate different data modalities. However, whereas linked ICA assumes that each data modality may be decomposed into a number of (shared) components, our model assumes that anatomical connectivity is the variable that is shared across modalities.

Our final model variant uses an informative prior which encodes the assumption that between-hemisphere connections are restricted to those between functionally homologous regions (cf. for example [66]). This is only one of many prior distributions that, depending on the research question and available background information, may be used to inform the connectivity estimates. As expected, the prior removes the negative partial correlations that are visible for contralateral connections in the other model variants. Indirectly, the prior also affects the partial correlations within hemispheres, as they become slightly lower in magnitude across the board. These results touch upon an unresolved issue in connectomics concerning the interpretation of negative (partial) correlations. It has been suggested that a substantial number of negative partial correlations are due to global signal regression and are therefore artifactual in nature rather than biological [67–69]. On the other hand, it has been shown that even without global signal regression, negative connections exist and these may even have biological meaning [70]. Although it is outside the scope of this paper to resolve this matter, we have shown that an informed prior may be used to encode such assumptions or correct for biases.

As our approach is Bayesian it directly allows for statistical inference, so that the uncertainty associated with our estimates may explicitly be quantified. In terms of a binary graph that indicates conditional (in)dependence, this expresses itself by providing an expectation of a connection rather than a point estimate. For partial correlations, the approach provides the supported distribution instead of a single value. These posterior distribution shapes reveal that none of the model variants are dominated by their mode. In particular for the original model the distributions are very broad and contain many unique models. Although a number of connections is consistently present, the conditional independence graphs vary substantially across subjects. In contrast, the data fusion approach and the informed prior result in distributions that are more tightly centered around the maximum a posteriori connectivity, yet even here there remains substantial support for alternative models. This has important implications for connectomics studies. These are typically aimed at obtaining a point estimate (which can often be interpreted as the mode of an implicit posterior distribution), so a substantial number of connections with significant support from the data will be excluded and spurious connections will be suggested. The widths of the posterior distributions strongly advocate a Bayesian approach, or at the very least point-estimated connectivity results should be treated with great care, e.g. by applying a bootstrapping procedure [71].

The main limitation of our study is one of scale. Bayesian inference has the drawback of being computationally demanding in approximating the posterior distributions, and although state-of-the-art machinery has been applied to make this process efficient, it remains impossible to apply the same methods to a large number of variables. Applying the models to large-scale data sets requires either more efficient implementations, e.g. by using GPU programming, or additional efficiency gains in the field of Gaussian graphical models.

Finally, a fundamental assumption in Gaussian graphical model estimation is that the functional data are normally distributed. Should this assumption fail, it may prove difficult to interpret the estimated connectivity. However, as discussed by [23], BOLD time series do tend to be mostly Gaussian.

The most pressing issue for future work is, as mentioned above, improving the methodology to handle a larger number of variables. However, a number of interesting research questions may be addressed even with a limited number of regions. For example, a model may be constructed that defines the BOLD time series to be generated by a mixture of partial correlation matrices, instead of a single one. By applying appropriate constraints, such as that consecutive datapoints are likely to be generated by the same connectivity matrix, this setup can be applied to differentiate experimental conditions based on their connectivity distributions [72]. Similarly, subjects may be assigned to either patients or healthy controls by defining a shared conditional independence graph for either group.

The data fusion approach may be extended to incorporate any number of imaging modalities, provided that a forward model can be constructed that shares at least one variable with the other modalities. For example, structural connectivity may inform functional connectivity estimated from MEG instead of or in addition to fMRI data [59].

Additional information may also be incorporated into the prior. This may be explicit evidence for (the absence of) a connection, e.g. tracer studies that reveal the presence of a fiber bundle can make particular connections more likely or, conversely, knowledge about white-matter lesions may preclude connections. Alternatively one could construct a prior in which the probability of a connection is a function of the distance between the corresponding end points.

In conclusion, the proposed Bayesian approach to functional connectivity has demonstrated that connectivity may be meaningfully divided into structure and strength. Several model variants have been discussed, each with their own characteristics. Application of the models has shown convincingly that multiple unique structures are possible given the same data. This illustrates the advantages of a Bayesian approach to connectivity, and provides a word of caution for traditional (regularized) maximum likelihood estimators.

## Supporting Information

S1 Text Markov chain Monte Carlo scheme for approximation of the posterior distributions. (PDF)

S2 Text Strategy for assessment of convergence of the MCMC samplers. (PDF)

S3 Text Influence of the prior. (PDF)

S1 Fig Performance of maximum likelihood estimates in recovery of simulation ground truth. (PDF)

S2 Fig A. Expected connectivity averaged over 20 subjects, using functional data. B. The corresponding standard deviation.
(PDF)

S3 Fig A. Expected connectivity averaged over 20 subjects, using functional and structural data. B. The corresponding standard deviation.
(PDF)

S4 Fig Expected connectivity averaged over 20 subjects, using functional data and a restrictive prior. (PDF)
