# Supplementary material for: Bayesian Estimation of Conditional Independence Graphs Improves Functional Connectivity Estimates
Source: PLoS Comput Biol. 2015 Nov 5;11(11):e1004534. doi: 10.1371/journal.pcbi.1004534 (PMC4634993; doi:10.1371/journal.pcbi.1004534)
Supplement: S1 Text — (PDF) [file pcbi.1004534.s001.pdf]

Bayesian estimation of conditional independence graphs improves functional connectivity estimates

Supporting Information S1

Max Hinne[1,2], Ronald J. Janssen[2], Tom Heskes[1] and Marcel A. J. van Gerven[2]

[1]Radboud University, Institute for Computing and Information Sciences, the Netherlands
[2]Radboud University, Donders Institute for Brain, Cognition and Behaviour, the Netherlands

Markov chain Monte Carlo for approximate inference

We approximate the posterior distributions using Metropolis Markov chain Monte Carlo. The procedure requires that we compute the acceptance ratio γ between the current state $(G, \mathbf{K})$ and the proposed state $(\tilde{G}, \tilde{\mathbf{K}})$. It is defined as the ratio of the posterior probabilities of the two states, i.e.

$$\gamma = \frac{P(\tilde{G}, \tilde{\mathbf{K}} \mid \mathbf{X})}{P(G, \mathbf{K} \mid \mathbf{X})} = \frac{P(\mathbf{X} \mid \tilde{G}, \tilde{\mathbf{K}})}{P(\mathbf{X} \mid G, \mathbf{K})} \frac{P(\tilde{G})}{P(G)} \ . \tag{1}$$

In general, the proposed state is accepted as a new sample with probability $\min(1, \gamma)$, otherwise the current state is stored instead. Until recently, this approach has proven impractical for this specific problem, as no efficient way was available to generate the proposed $\tilde{\mathbf{K}}$. Instead, a block Gibbs sampler that updates $\mathbf{K}$ according to either the edges of G [Wang and Li, 2012] or its clique decomposition [Piccioni, 2000] was used, but this is a computationally demanding solution. Significant improvement was obtained by the introduction of a direct sampler for the G-Wishart distribution [Lenkoski, 2013]. Together with a reversible-jump setup [Green, 1995], this resulted in a much faster way of computing (1), as described in [Lenkoski, 2013]. Additional efficiency was gained in recent work, by exploiting the analytical properties of the conditional Bayes factor $P(\mathbf{X} \mid G, \mathbf{K})$ to compute γ more efficiently [Hinne et al., 2014]. The corresponding algorithm is used in this paper.

When the data from probabilistic tractography are added, the acceptance ratio becomes

$$\gamma' = \frac{P(\mathbf{X} \mid \tilde{G}, \tilde{\mathbf{K}})}{P(\mathbf{X} \mid G, \mathbf{K})} \frac{P(\mathbf{S} \mid \tilde{G})}{P(\mathbf{S} \mid G)} \frac{P(\tilde{G})}{P(G)} \ . \tag{2}$$

As $\mathbf{X}$ and $\mathbf{S}$ are conditionally independent given G, computing γ' consists of multiplying γ with the acceptance ratio of the structural model, which is described in detail in [Hinne et al., 2013].

References

P J Green. Reversible jump Markov chain Monte Carlo computation and Bayesian model determination. *Biometrika*, 82:711–732, 1995.

M Hinne, T Heskes, CF Beckmann, and M A J van Gerven. Bayesian inference of structural brain networks. *NeuroImage*, 66C:543–552, 2013.

M Hinne, A Lenkoski, T Heskes, and M A J van Gerven. Efficient sampling of Gaussian graphical models using conditional Bayes factors. *Stat*, 3:326–336, 2014.

A Lenkoski. A direct sampler for G-Wishart variates. *Stat*, 2(1):119–128, 2013.

M Piccioni. Independence structure of natural conjugate densities to exponential families and the Gibbs sampler. *Scandinavian Journal of Statistics*, 27:111–127, 2000.

H Wang and S Z Li. Efficient Gaussian graphical model determination under G-Wishart distributions. *Electronic Journal of Statistics*, 6:168–198, 2012.
