# Supplementary material for: Bayesian Estimation of Conditional Independence Graphs Improves Functional Connectivity Estimates
Source: PLoS Comput Biol. 2015 Nov 5;11(11):e1004534. doi: 10.1371/journal.pcbi.1004534 (PMC4634993; doi:10.1371/journal.pcbi.1004534)
Supplement: S2 Text — (PDF) [file pcbi.1004534.s002.pdf]

Bayesian estimation of conditional independence graphs improves functional connectivity estimates

Supporting Information S2

Max Hinne[1,2], Ronald J. Janssen[2], Tom Heskes[1]and Marcel A. J. van Gerven[2]

[1]Radboud University, Institute for Computing and Information Sciences, the Netherlands
[2]Radboud University, Donders Institute for Brain, Cognition and Behaviour, the Netherlands

Convergence

For each distribution, three parallel chains were executed until convergence. Convergence was assessed by computing the potential scale reduction factor (PSRF) [Gelman and Rubin, 1992], which can be understood as a ratio between intra-chain variance and inter-chain variance. Heuristically, we assumed convergence for connectivity when for each edge (i, j) and each element k_{ij} the PSRF dropped below 1.1. For the first model, convergence was attained within 20,000 samples. The multimodal approach proved substantially slower to converge, requiring between 50,000 and 200,000 samples. Four subjects contained edges and elements in the precision matrix that did not fully converge within reasonable time. However, for these subjects the chains did reach convergence in terms of the network density of G and the matrix determinant of $\mathbf{K}$, which motivated us to treat them the same as the other subjects. The last model with a more specific prior decreases the number of free parameters, which makes the convergence substantially faster; convergence was attained within 10,000 samples.

The computation speed of a single sample is determined by an iteration over $p(p-1)/2$ elements and computing two Cholesky decompositions for each element, resulting in a total time complexity of $O(p^5)$. Although an efficient implementation in C++ using BLAS [Blackford et al., 2002] and LAPACK [Anderson et al., 1999] was used, the complexity of the algorithm makes the approach infeasible for use with a large number of variables. To demonstrate this, Fig 1 shows for different values of p the corresponding computation time of a single sample, using a single 2.60GHz processor. Note that in the main text, the simulations consisted of 5, 10, 15 or 50 nodes, and the empirical data contained 14 subcortical areas.

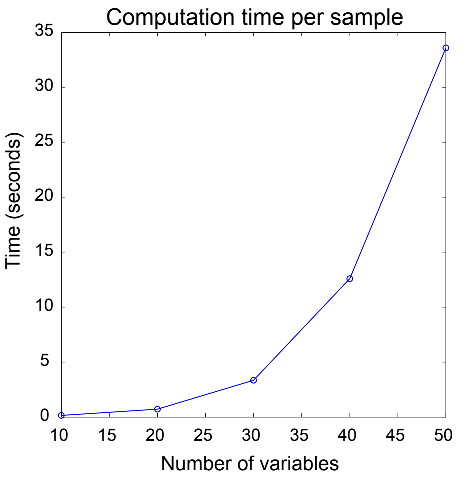

Figure 1. Computation time per sample (in seconds) for different numbers of variables.

References

E Anderson, Z Bai, C Bischof, S Blackford, J Demmel, J Dongarra, J Du Croz, A Greenbaum, S Hammarling, A McKenney, and D Sorensen. *LAPACK Users' Guide*. Society for Industrial and Applied Mathematics, Philadelphia, PA, third edition, 1999. ISBN 0-89871-447-8 (paperback).

LS Blackford, J Demmel, J Dongarra, I Duff, S Hammarling, G Henry, M Heroux, L Kaufman, A Lumsdaine, R Petitet, R Pozo, K Remington, and RC Whaley. An updated set of basic linear algebra subprograms (blas). *AMC Trans Math Soft*, 28(2):135–151, 2002.

A Gelman and D B Rubin. Inference from iterative simulation using multiple sequences. *Statistical Science*, 7(4):457–472, 1992.
