# Supplementary material for: Bayesian Estimation of Conditional Independence Graphs Improves Functional Connectivity Estimates
Source: PLoS Comput Biol. 2015 Nov 5;11(11):e1004534. doi: 10.1371/journal.pcbi.1004534 (PMC4634993; doi:10.1371/journal.pcbi.1004534)
Supplement: S3 Text — (PDF) [file pcbi.1004534.s003.pdf]

Bayesian estimation of conditional independence graphs improves functional connectivity estimates

Supporting Information S3

Max Hinne[1,2], Ronald J. Janssen[2], Tom Heskes[1]and Marcel A. J. van Gerven[2]

[1]Radboud University, Institute for Computing and Information Sciences, the Netherlands
[2]Radboud University, Donders Institute for Brain, Cognition and Behaviour, the Netherlands

Prior influence

Throughout our experiments we use a Bernoulli distribution with parameter θ to define the probability of an edge. In the prior, we consider all edges to be conditionally independent, so we have

$$P(G \mid \boldsymbol{\Theta}) = \prod_{i<j} \theta_{ij}^{g_{ij}} (1 - \theta_{ij})^{1-g_{ij}} \ , \tag{1}$$

with $\Theta = (\theta_{ij})_{i<j}$. Conform Hinne et al. [2013], we adopt $\forall_{i,j}[\theta_{ij} = 0.5]$. Note that $\mathbb{E}[d \mid \theta] = \theta$, with $d(G)$ the density of G, i.e. the fraction of present edges.

To analyze the influence of the prior, we repeated the connectivity estimation procedure for one subject, by collecting 1 000 samples with $\forall_{i,j}[\theta_{ij} = \{0.1, \ldots, 0.9\}$. The extreme values $\theta = \{0.0, 1.0\}$ have been excluded as in these cases the prior would fully determine the posterior outcome (see also the discussion of the informed prior in the main text). As shown in Fig. 1, the mean posterior network density is in the range $[0.52, 0.75]$, a much smaller range than the prior promotes. This indicates that although the effect of the prior cannot be ignored entirely, the posterior and prior network densities are far apart. This allows us to conclude that the posterior network density is dominated by the likelihood.

References

M Hinne, T Heskes, CF Beckmann, and M A J van Gerven. Bayesian inference of structural brain networks. *NeuroImage*, 66C:543–552, 2013.

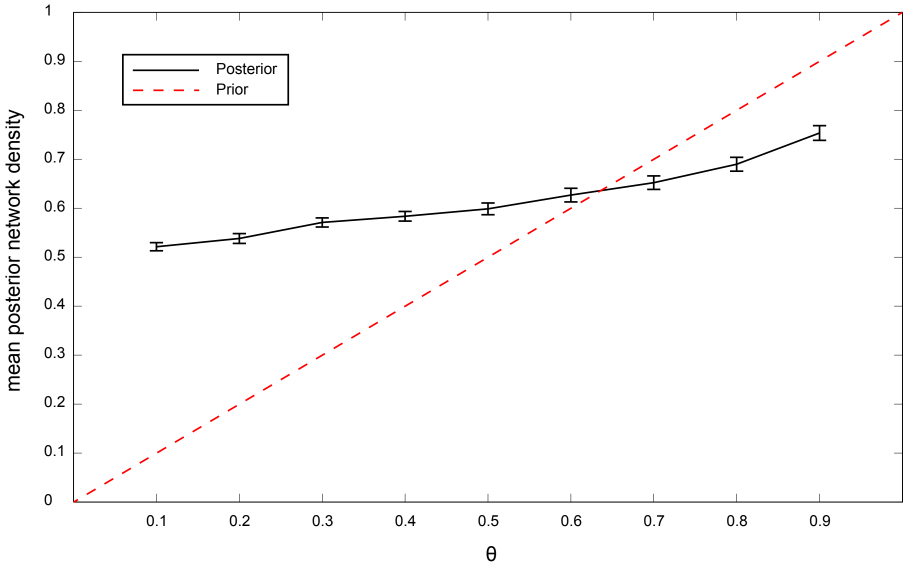

Figure 1. The mean posterior network density as a function of the Bernoulli parameter θ. The prior network density is fully determined by θ and is indicated with the red dotted line. Error bars indicate one standard deviation of the posterior network density.
