# Supplementary material for: Bayesian Estimation of Conditional Independence Graphs Improves Functional Connectivity Estimates
Source: PLoS Comput Biol. 2015 Nov 5;11(11):e1004534. doi: 10.1371/journal.pcbi.1004534 (PMC4634993; doi:10.1371/journal.pcbi.1004534)
Supplement: S1 Fig — (PDF) [file pcbi.1004534.s004.pdf]

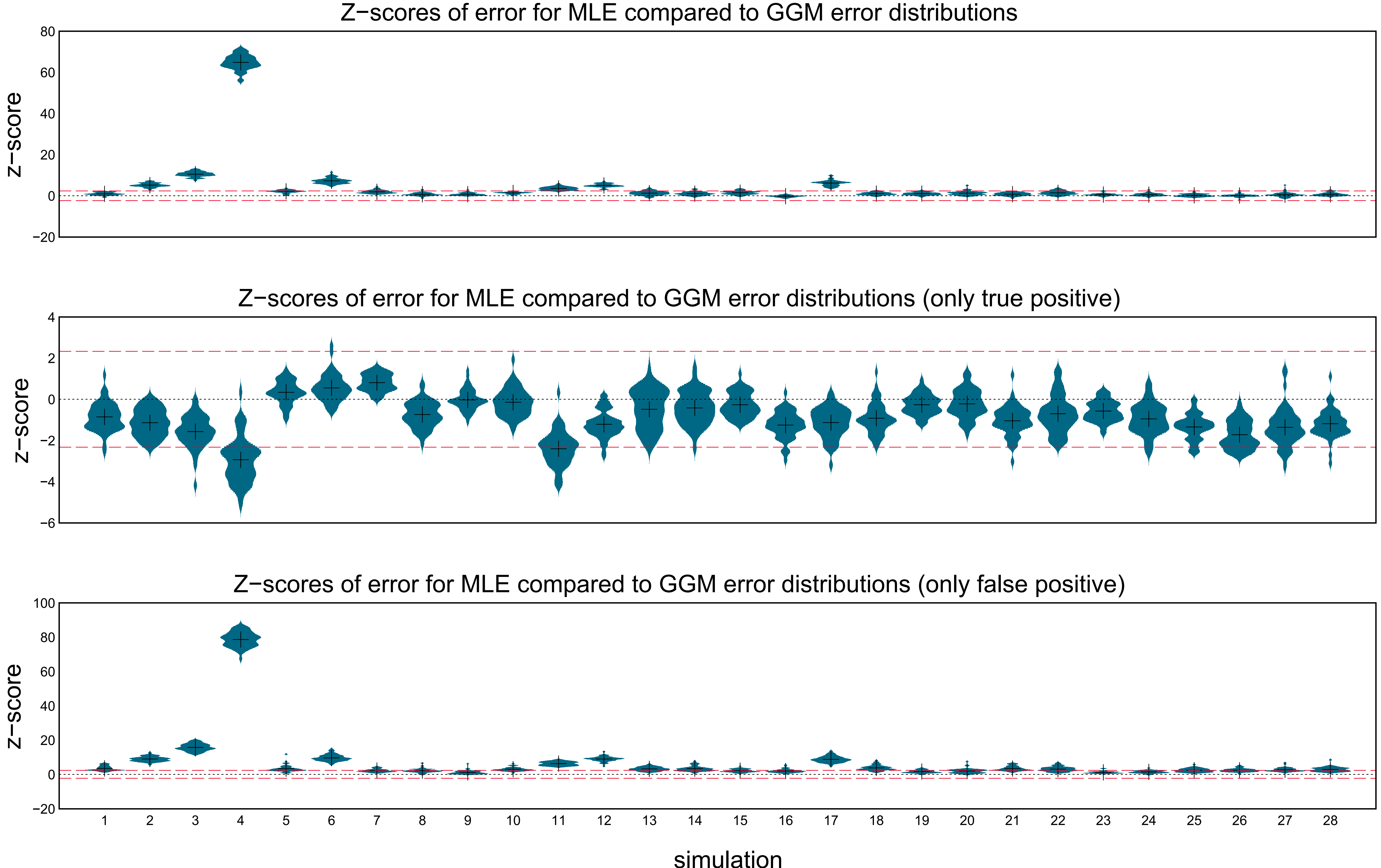
Z−scores of error for MLE compared to GGM error distributions
z−score
80
60
40
20
0
−20
Z−scores of error for MLE compared to GGM error distributions (only true positive)
z−score
4
2
0
−2
−4
−6
Z−scores of error for MLE compared to GGM error distributions (only false positive)
z−score
100
80
60
40
20
0
−20
1 2 3 4 5 6 7 8 9 10 11 12 13 14 15 16 17 18 19 20 21 22 23 24 25 26 27 28
simulation
