# Supplementary figures and images for: Bayesian Estimation of Conditional Independence Graphs Improves Functional Connectivity Estimates
Source: PLoS Comput Biol. 2015 Nov 5;11(11):e1004534. doi: 10.1371/journal.pcbi.1004534 (PMC4634993; doi:10.1371/journal.pcbi.1004534)

**A**

Independence structure

Partial correlations

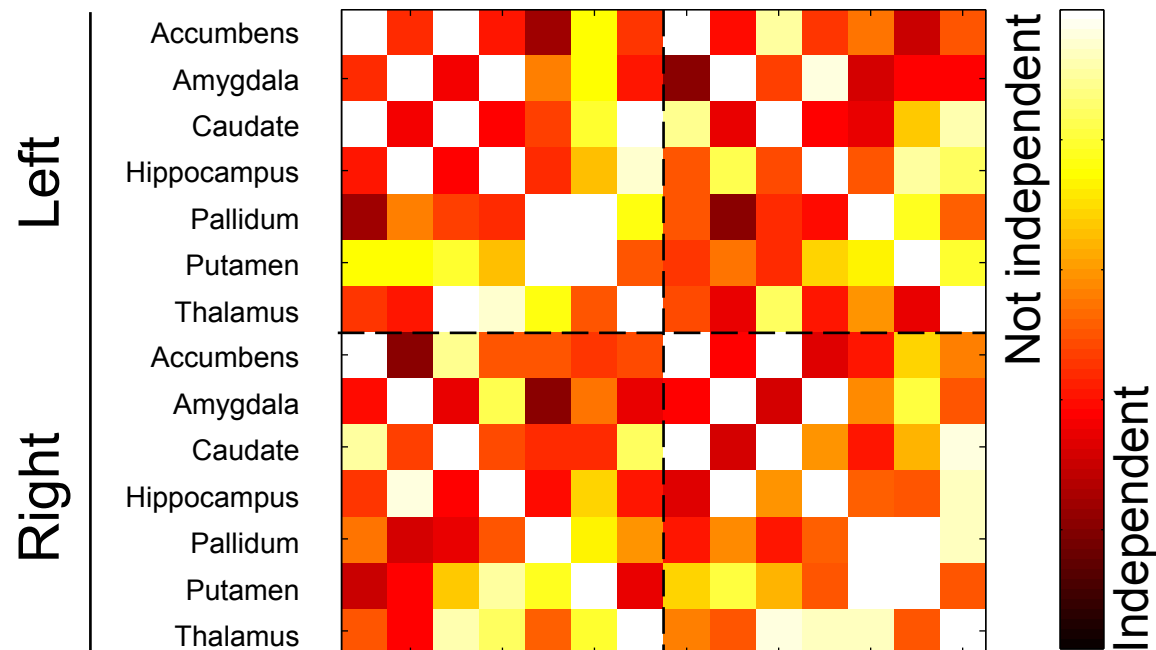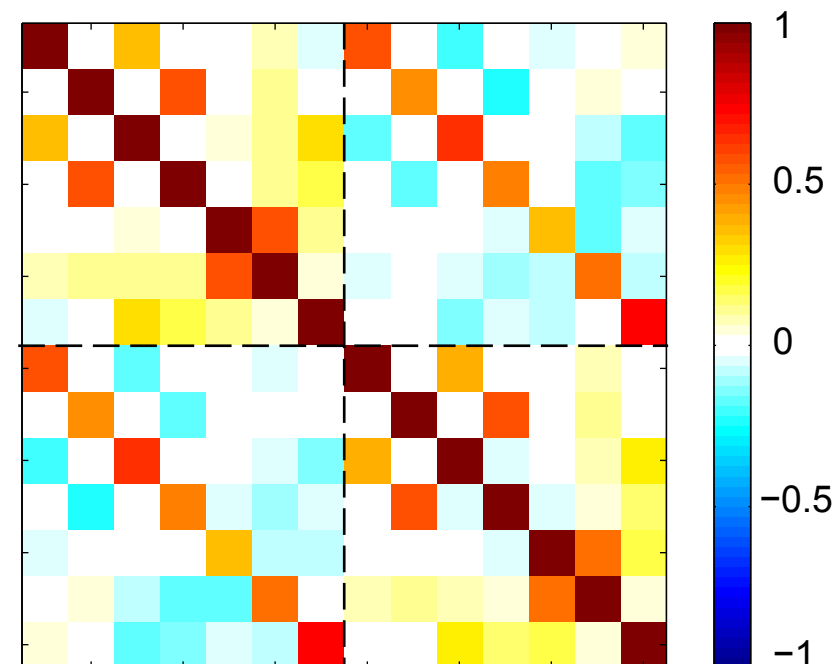**B**

Standard deviation

Standard deviation

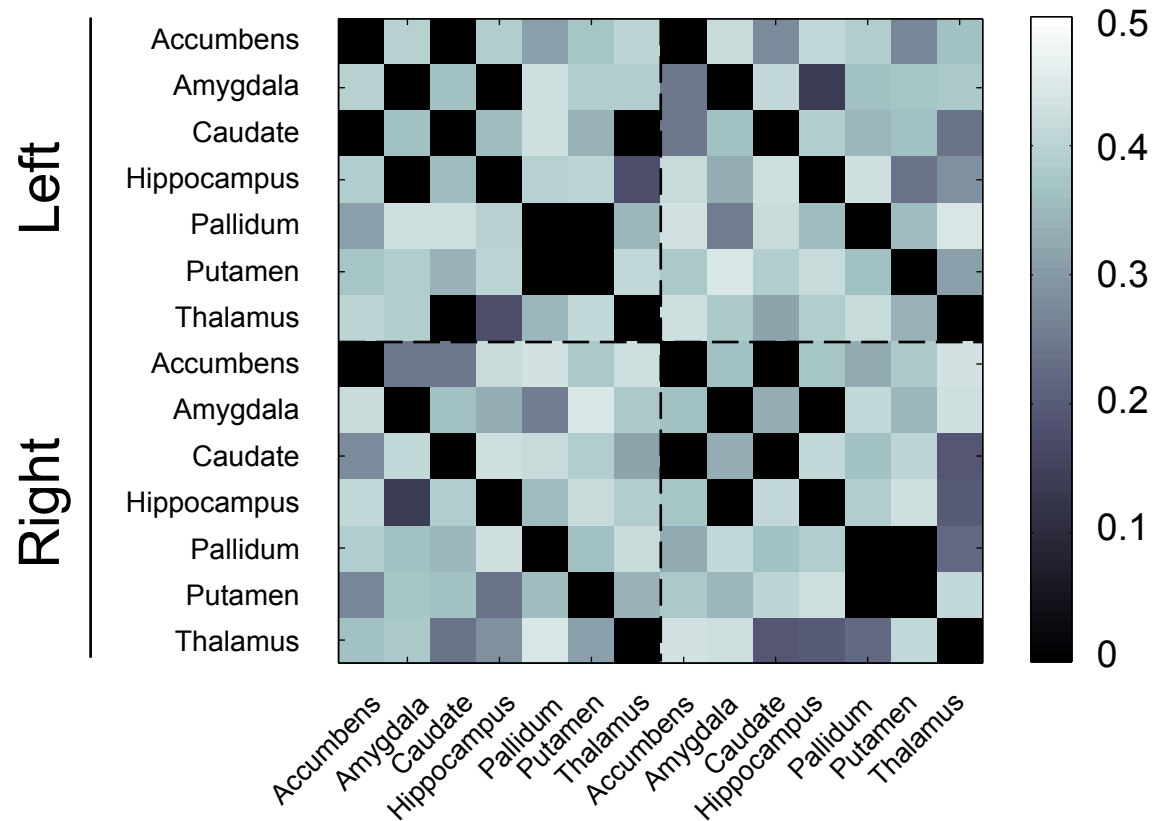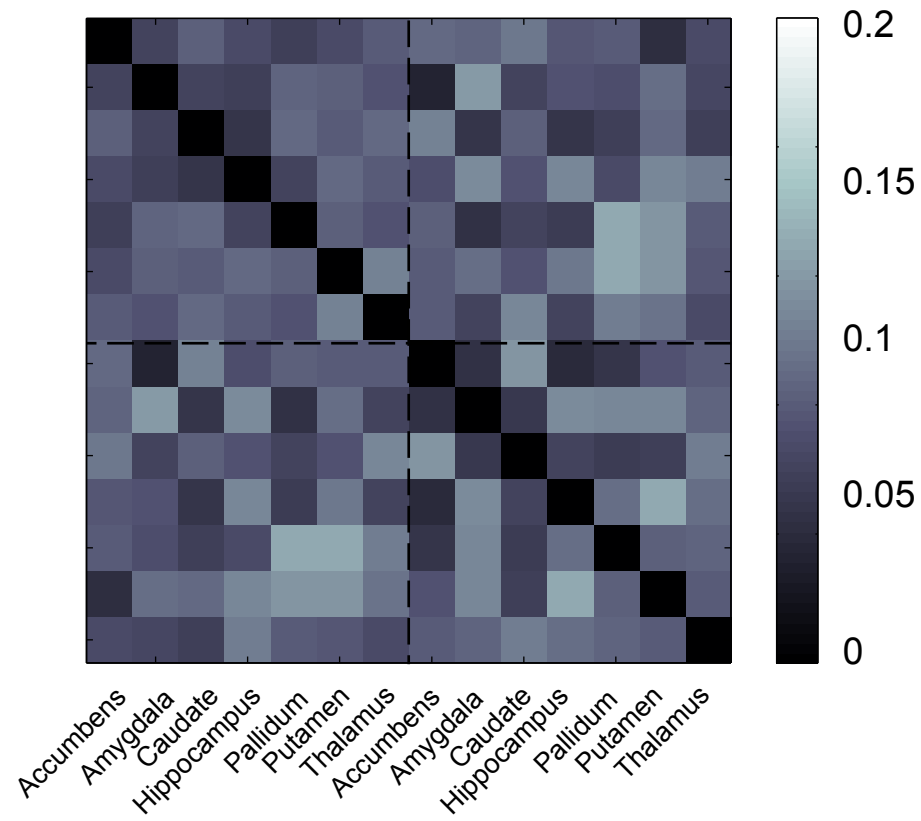

Supplement: S2 Fig — A. Expected connectivity averaged over 20 subjects, using functional data. B. The corresponding standard deviation. (PDF) [file pcbi.1004534.s005.pdf]

**A**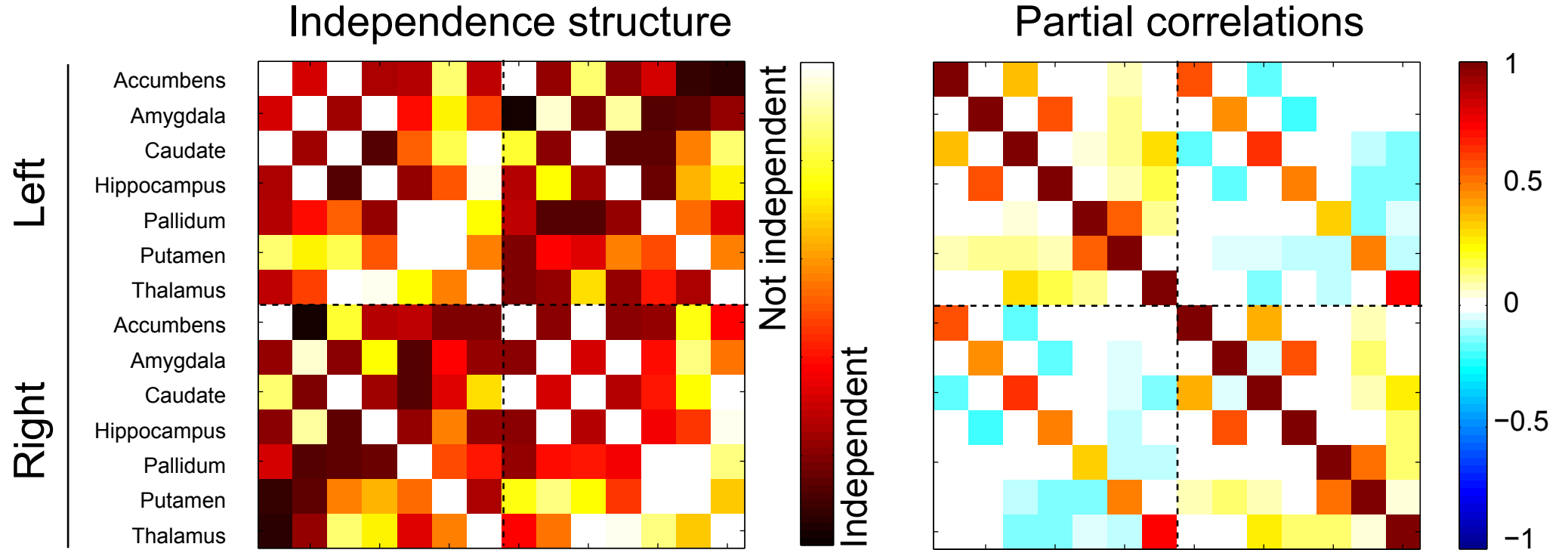**B**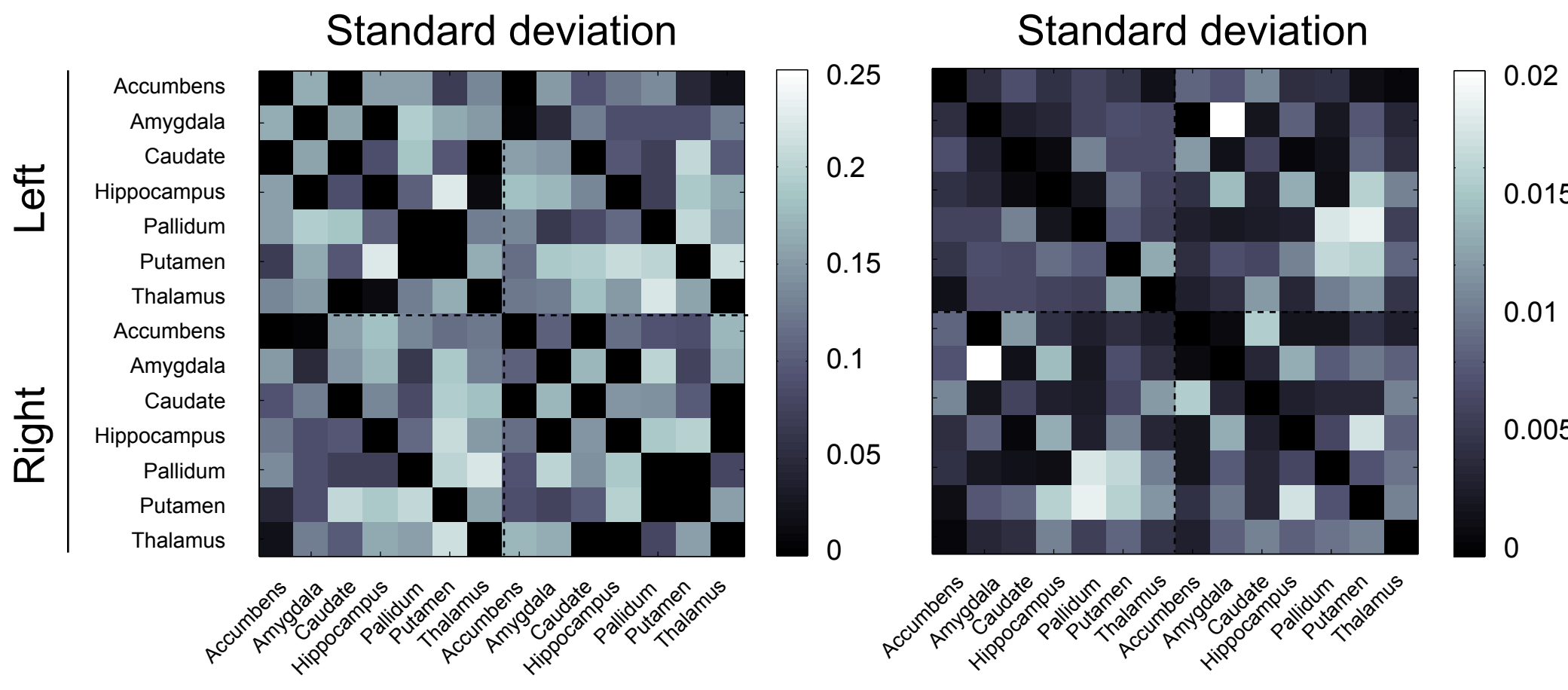

Supplement: S3 Fig — A. Expected connectivity averaged over 20 subjects, using functional and structural data. B. The corresponding standard deviation. (PDF) [file pcbi.1004534.s006.pdf]
