# Supplementary material for: Bayesian Estimation of Conditional Independence Graphs Improves Functional Connectivity Estimates
Source: PLoS Comput Biol. 2015 Nov 5;11(11):e1004534. doi: 10.1371/journal.pcbi.1004534 (PMC4634993; doi:10.1371/journal.pcbi.1004534)
Supplement: S4 Fig — (PDF) [file pcbi.1004534.s007.pdf]

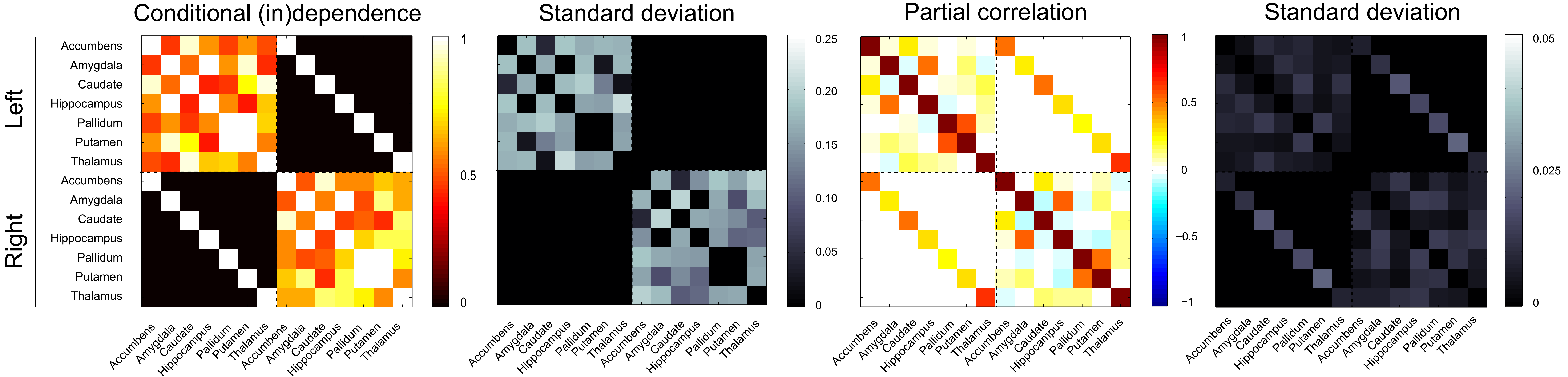

Conditional (in)dependence
Standard deviation
Partial correlation
Standard deviation
Left
Right
Accumbens
Amygdala
Caudate
Hippocampus
Pallidum
Putamen
Thalamus
1
0.5
0
0.25
0.20
0.15
0.10
0.05
0
1
0.5
0
−0.5
−1
0.05
0.025
0
